# Uncertainty-Aware General Gaze Following via Circular Direction Distribution Learning and Probabilistic Gaze Geometry Modeling

**DOI:** 10.3390/jemr19040088

**Published:** 2026-08-13

**Authors:** Yanzhao Li, Jin Li, Xiaona Zhang, Hong Liang

**Affiliations:** 1 College of Intelligent Systems Science and Engineering, Harbin Engineering University, Harbin 150001, China; 2 School of Economics and Management, Harbin Engineering University, Harbin 150001, China

**Keywords:** general gaze following, gaze target localization, visual attention, social attention, directional uncertainty, Circular Direction Distribution Learning, Probabilistic Gaze Geometry Modeling

## Abstract

General gaze following aims to infer the region attended to by a person within a natural scene and offers a computational perspective on visual attention and social scene understanding. Existing direction-guided methods usually reduce gaze direction to a single vector or spatial mask. This deterministic treatment can obscure directional ambiguity, discard coexisting candidate directions, and propagate early estimation errors to gaze target localization when head cues are weak or multiple targets are plausible. To address these limitations, we propose an uncertainty-aware framework based on Circular Direction Distribution Learning (CDDL) and Probabilistic Gaze Geometry Modeling (PGGM). CDDL represents gaze direction as a 72-bin circular probability distribution under von Mises soft supervision, thereby preserving neighboring directional hypotheses before target localization. Rather than predicting a target space distribution directly, PGGM aggregates the direction distribution into 18 groups and projects the retained hypotheses into cone-like spatial probability fields, allowing spatial tolerance to expand with distance while preserving a channel-wise direction-to-region representation. The full probability volume is fused with the RGB scene image at the input level and processed by a ResNet-50-FPN network for gaze heatmap prediction. Controlled experiments on GazeFollow demonstrate competitive localization and support the contribution of direction-distribution learning and channel-wise geometric projection. Zero-shot transfer, dataset-level uncertainty and failure analyses, efficiency measurements, and qualitative results further indicate interpretable behavior together with domain-dependent and computational trade-offs.

## 1. Introduction

Gaze is a fundamental social signal that helps people infer others’ attention, intentions, and interactional goals. The ability to follow another person’s gaze supports joint attention, social communication, and the interpretation of behavior in everyday scenes [1,2,3]. From a computational perspective, general gaze following aims to infer the region attended to by a selected person in a natural image. This task provides a person-conditioned account of visual attention, in which the target region is determined not only by scene appearance but also by the observed person’s directional cues and social context. Psychological accounts of shared attention place gaze perception and gaze following within a broader system that links the detection of another person’s attentional direction to joint attention and higher-level social cognition [4]. This perspective motivates computational models that preserve uncertainty when another person’s attentional focus cannot be inferred unambiguously from visual evidence.

Whereas eye tracking and appearance-based gaze estimation directly characterize an observer’s oculomotor behavior or visual axis, image-based general gaze following addresses a complementary third-person inference problem. It estimates where a depicted person is likely to be looking by integrating head appearance, head location, scene structure, and contextual cues [5,6,7]. This distinction is particularly important in unconstrained images, where the eyes may be invisible, the face may be partially occluded, and the head may be observed from the side or back. Under such conditions, direct ocular evidence is unavailable, and the attended region must be inferred from weaker but complementary visual cues. Experimental studies of facially cued orienting further show that facial and gaze-related signals can modulate overt attention and saccadic trajectories in human observers [8]. These observer-centered effects establish the behavioral relevance of gaze cues, but they remain distinct from the present task, which infers the attended region of a depicted person from third-person image evidence.

General gaze following was established as a large-scale visual inference task by [9], which introduced the GazeFollow benchmark and demonstrated the importance of jointly modeling head-related cues and scene saliency. Subsequently, Ref. [10] introduced a two-stage formulation that first estimates gaze direction from head appearance and head location and then uses direction fields to guide heatmap-based target localization. This design provides a more explicit connection between directional evidence and candidate target regions. However, the intermediate direction is still represented as a single deterministic vector, such that directional uncertainty is collapsed before it can be exploited by the downstream heatmap network. Ref. [11] further incorporated human-centric relationships and semantic context, demonstrating that gaze target inference benefits from both geometric and relational information.

Recent work has substantially expanded the capability of gaze target estimation through end-to-end detection, Transformer-based interaction modeling, multi-person reasoning, and large-scale visual representations. For example, Ref. [12] formulates gaze following as a joint human–gaze target detection task; Ref. [13] models multiple people and their gaze targets simultaneously; and [14] exploits interaction features from Vision Transformers. More recent studies have explored large-scale learned encoders [15], multi-view information for resolving occlusion and target ambiguity [16], and promptable concept-based gaze target estimation [17]. These advances demonstrate that gaze following benefits from richer scene representations and semantic reasoning. However, stronger visual representations do not by themselves resolve a fundamental ambiguity in third-person gaze inference: head-related evidence may support a range of nearby viewing directions rather than a single precise direction. This issue becomes particularly relevant when the face is partially visible, the person is viewed from the side or back, or several candidate objects are compatible with the same coarse head orientation [18,19].

The distinction between scene saliency and person-conditioned attention is also central to gaze following. A visually salient region may attract a generic observer, but it is not necessarily the object attended to by a particular person in the image. Conversely, a person may attend to a visually inconspicuous object when it is relevant to an ongoing action or social interaction. Recent eye-movement research continues to show that spatial organization, task relevance, and cognitive structure can shape visual attention beyond simple stimulus saliency [20,21,22]. For gaze following, this implies that target prediction should not rely solely on global saliency; it should preserve person-specific directional evidence while allowing scene context to discriminate among multiple plausible target regions.

Accordingly, the central question addressed in this study is not only how to improve gaze target localization through a stronger visual backbone, but also how to preserve and use directional ambiguity before it is collapsed into a single spatial prior. In natural scenes, facial details may be unavailable, head orientation may be only weakly informative, and several nearby objects may remain compatible with the same coarse viewing direction. In addition, gaze target annotations may exhibit observer-dependent variation when the attended object is small, distant, spatially extended, or semantically ambiguous [23]. A deterministic gaze vector or a single gaze cone can provide useful geometric guidance, but it may prematurely discard neighboring and competing directional hypotheses. Once this information is removed, an early directional error can be directly propagated to the subsequent heatmap prediction stage. A suitable intermediate representation should therefore preserve directional belief across neighboring orientations and retain multiple plausible direction-to-region hypotheses before integrating them with scene appearance.

To address this issue, we propose an uncertainty-aware general gaze-following framework based on Circular Direction Distribution Learning (CDDL) and Probabilistic Gaze Geometry Modeling (PGGM). CDDL replaces deterministic direction regression with 72-bin circular distribution learning under von Mises soft supervision. Rather than treating only one direction as valid, this formulation represents the directional belief over the full image-space circle and preserves uncertainty around the most likely direction. PGGM then aggregates the learned direction distribution into 18 direction groups and maps them into cone-like spatial probability fields. The corresponding fields are retained as a channel-wise probability volume, allowing multiple directional hypotheses to remain distinguishable during gaze target prediction.

The full PGGM volume is concatenated with the RGB scene image at the input level and processed by a ResNet-50-FPN network for heatmap prediction. This image-level early fusion enables the network to jointly exploit scene appearance, person-conditioned geometry, and directional uncertainty from the first stages of feature extraction. The proposed framework is designed as a computational account of how uncertain directional evidence can be transformed into spatially structured target hypotheses, while retaining an interpretable direction-to-region reasoning process.

The main contributions of this study are summarized as follows:

1. We propose Circular Direction Distribution Learning, which represents gaze direction as a circular probability distribution rather than a deterministic regression target. The proposed formulation preserves directional ambiguity among neighboring directions without requiring additional annotations.
2. We propose Probabilistic Gaze Geometry Modeling, which transforms the learned direction distribution into a set of spatial probability fields. The cone-like formulation models distance-dependent uncertainty expansion and preserves multiple directional hypotheses through a channel-wise probability volume.
3. We introduce an image-level early-fusion strategy that integrates the full PGGM volume with the RGB scene image before heatmap prediction, enabling the network to jointly learn visual appearance and probabilistic geometric evidence.
4. We conduct systematic experiments on GazeFollow to evaluate circular direction learning, probabilistic field construction, and image-level fusion. The results show that the proposed framework provides competitive gaze target localization performance while maintaining an interpretable direction-to-region reasoning process.

## 2. Related Work

### 2.1. General Gaze Following and Person-Conditioned Visual Attention

Gaze following and eye tracking are closely related but address different levels of visual attention analysis. Eye tracking and appearance-based gaze estimation typically measure or estimate an observer’s viewing direction from ocular or facial signals. By contrast, image-based general gaze following infers the attended region of a depicted person from third-person visual evidence, including head appearance, head position, scene content, and social context. Thus, it provides a computational account of person-conditioned attention in natural scenes rather than a direct measurement of oculomotor behavior [24,25]. Recent overviews of eye-tracking research have also highlighted the growing role of deep learning, multimodal interaction, and computational gaze analysis in understanding visual attention and human-centered interaction [26,27].

The general gaze-following task was systematically formulated by [9], which introduced the GazeFollow benchmark and demonstrated that gaze target inference requires both person-related cues and scene-level information. The method combined a gaze pathway with a saliency pathway, thereby establishing the importance of integrating directional evidence with candidate regions in the image. Later, Ref. [10] introduced a two-stage formulation that first estimates gaze direction from head appearance and head position and then uses direction fields to guide heatmap-based gaze target localization. This formulation made the directional cue more explicit and provided an interpretable bridge between head-related evidence and spatial target prediction.

Person-conditioned attention differs fundamentally from generic visual saliency. Classical and deep saliency models characterize generic fixation patterns through bottom-up contrast and learned scene representations [28,29,30,31,32]. A region that is visually conspicuous for a generic observer is not necessarily the region attended to by a particular person, whereas an inconspicuous object may become the gaze target because of its relevance to an ongoing action or social interaction. Eye movement research similarly indicates that visual attention is shaped by spatial organization, task relevance, and cognitive context, rather than by stimulus saliency alone [21,22]. Accordingly, gaze-following models need to retain person-specific directional evidence while using scene context to distinguish among plausible target regions.

### 2.2. Contemporary Gaze Target Estimation and Human–Scene Reasoning

Subsequent gaze-following studies have incorporated richer contextual cues to improve target localization. Ref. [11] introduced human-centric relationship reasoning by integrating head information, scene saliency, and object-level contextual cues. This line of work showed that gaze target estimation benefits from modeling the semantic relationships between a person and surrounding objects or other people [33]. More generally, relation networks and scene graph models have shown that pairwise relations, scene-level context, and object–context structure can improve high-level visual inference [34,35,36,37,38]. However, relation-based approaches may depend on object proposals, detection quality, or additional semantic modules, which can increase system complexity.

Recent methods have increasingly adopted stronger visual encoders and global interaction mechanisms. Ref. [13] extends gaze following to multi-person scenes through a Transformer-based formulation, while [14] exploits interaction features within pre-trained Vision Transformers to model human–scene relationships. Large-scale learned encoders have also been explored in [15], demonstrating the value of transferable visual representations for gaze target estimation. In addition, recent work has investigated multi-view observations for resolving occlusion and target ambiguity [16], limited-label learning with saliency guidance [39], and concept-driven target reasoning [17]. Recent work has also examined vision-language models as a zero-shot source of contextual cues and has recast gaze target prediction as semantic target identification [40,41]. Complementary studies have incorporated conversational audio cues, limited-label supervision, and explicit head–target association to enrich gaze target inference under different input and annotation settings [18,39,42].

Another emerging direction concerns social gaze and shared attention. Ref. [43] jointly considers individual gaze target prediction and social gaze relationships, illustrating that gaze understanding can extend beyond single-person localization toward the interpretation of inter-personal attentional structure [19,44]. Related benchmark work has further extended gaze target research to child–adult interactions, where shared attention and gaze behavior are central to social development and interaction analysis [45]. These advances enrich the semantic and relational modeling of gaze behavior. Nevertheless, their principal emphasis is commonly placed on stronger scene representations, richer modalities, or broader interaction reasoning. The treatment of directional uncertainty as an explicit intermediate representation remains comparatively less developed.

### 2.3. Directional Uncertainty and Interpretable Spatial Gaze Geometry

Directional modeling provides an interpretable connection between head-related evidence and gaze target localization. In direction field-based frameworks, a predicted gaze direction is transformed into a spatial prior, allowing a heatmap network to search for targets along a geometrically plausible region [10]. Related research has further explored the role of geometric constraints in more complex spatial settings, including 360-degree images [46] and depth-aware gaze cone construction [47]. These studies demonstrate that geometric priors can effectively constrain gaze target search while remaining interpretable. Parallel studies have further used gaze-following labels to strengthen unconstrained three-dimensional gaze estimation and gaze-aware three-dimensional context encoding [48,49]. Temporal gaze-following studies have further modeled dynamic head–scene interactions and cross-frame geometry in videos [50,51].

However, many direction-guided methods ultimately reduce the directional cue to a single vector, a single cone, or a single spatial mask. Such a deterministic representation can be restrictive in natural scenes. When facial details are unavailable, the head is seen from the side or back, or multiple candidate objects lie near the same coarse viewing direction, the evidence may support several neighboring or competing directional hypotheses. Furthermore, GazeFollow annotations are collected from third-person observers, so target locations may exhibit variation when the attended object is small, distant, spatially extended, or semantically ambiguous [23]. In these cases, prematurely selecting only one direction can lead to overconfident spatial priors and amplify early estimation errors during heatmap prediction.

A probabilistic directional representation offers a suitable alternative. Rather than treating direction prediction as a single-point estimation problem, it can preserve the distribution of directional belief across neighboring orientations and retain competing hypotheses for subsequent scene-based disambiguation. This representation should be distinguished from a direct measurement of human eye movement variability: it describes uncertainty in computational third-person inference under incomplete visual evidence. Building on this perspective, the present study models gaze direction as a circular probability distribution and transforms it into a channel-wise spatial probability volume. The resulting formulation preserves directional ambiguity before integrating it with scene appearance, thereby providing an interpretable direction-to-region mechanism for gaze target localization.

## 3. Methods

**Figure 1:**
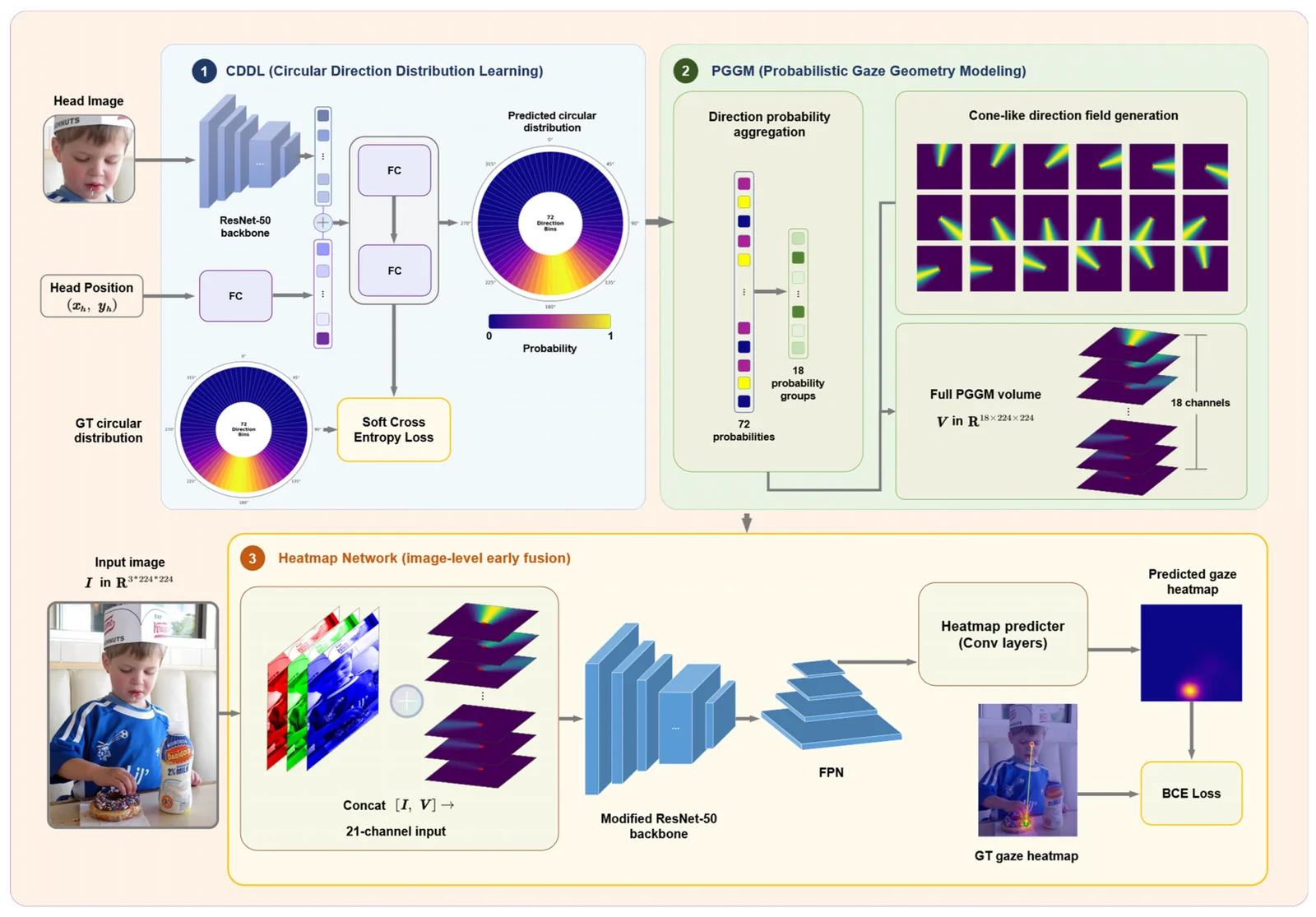
Overview of the CDDL–PGGM framework. CDDL predicts a 72-bin circular direction distribution from the head crop and head position. PGGM aggregates the distribution into 18 groups and constructs an 18-channel Direction Probability Volume using CGF. Concatenation with the RGB image produces the 21-channel input to the ResNet-50-FPN Heatmap Prediction Network.

Figure 1 summarizes the CDDL–PGGM framework. Given a target person, CDDL estimates a circular gaze direction distribution from the head crop and normalized head position. PGGM aggregates this distribution into direction groups and projects each group into a probability-weighted spatial field. The resulting Direction Probability Volume is concatenated with the RGB image and processed by a ResNet-50-FPN Heatmap Prediction Network. The following subsections define CDDL, PGGM, the heatmap network, and their stage-specific objectives.

### 3.1. Circular Direction Distribution Learning Network (CDDL)

CDDL models gaze direction as a circular distribution using the standard GazeFollow annotations. We first define the image space angle convention and the von Mises soft label, and then describe the direction network.

#### 3.1.1. The Definition of the Image Space Coordinate System

The circular image space direction is discretized into 72 bins of 5°. This resolution provides finer supervision than a 36-bin partition while avoiding the sample sparsity of substantially finer partitions such as 360 bins [52]. It is treated as a practical design resolution rather than a theoretically unique optimum.

For the direction definition in the image coordinate system, we take the head position (the head center) as the origin. The direction of 0° is defined as the upward direction, and the angle increases clockwise. This definition is consistent with image space intuition and is well aligned with visual perception and gaze cone visualization. Based on this convention, the 72 bins are defined as follows: bin 1 [0°,5°), bin 2 [5°,10°) … bin 72 [355°,360°). For an arbitrary gaze vector $\left(d_{x},d_{y}\right)$, the corresponding angle is computed as:

(1) $$\theta =atan2\left(d_{x},-d_{y}\right)\times \frac{180}{\pi },$$

if θ<0, it is further normalized by $\theta \leftarrow \theta +360^{{}^{\circ}}$.

#### 3.1.2. Soft Label Generation

The target label in our formulation is expected to be a circular probability distribution, denoted as:

(2) $$p=\left[p_{1},p_{2},\ldots ,p_{72}\right],$$

where $p_{i}\geq 0$ and $\sum_{i=1}^{72}p_{i}=1$.

This implies that, for a given gaze direction, the network should no longer assume that there exists only a single correct direction. Instead, directions in the neighborhood of the ground truth direction should also be regarded as plausible. Such a formulation is beneficial for handling annotation ambiguity, since gaze ambiguity is inherently continuous in the angular domain.

A conventional way to generate soft labels is Gaussian label smoothing, which can be formulated as:

(3) $$p_{i}\propto \exp\left(-\frac{{\left(\theta_{i}-\theta_{gt}\right)}^{2}}{2\sigma^{2}}\right).$$

However, this formulation is not circular. For example, under our direction definition, when the ground truth direction is $\theta_{gt}=0{}^{\circ}$, and the predicted direction is $\theta_{i}=359{}^{\circ}$, the prediction should be considered very close to the ground truth. Nevertheless, Gaussian label smoothing treats their angular difference as 359°, thereby regarding the prediction as almost completely incorrect. This is because the direction space in our problem is essentially circular, rather than a standard Euclidean space. Therefore, circular modeling is required.

To this end, we employ a circular distribution to construct the ground truth label distribution. Specifically, we adopt the von Mises distribution, which can be regarded as the Gaussian distribution defined on a circular domain [53]. The soft label for the i-th directional bin is defined as:

(4) $$p_{i}=\frac{\exp\left(\kappa \cos\left(\theta_{i}-\theta_{gt}\right)\right)}{\sum_{j=1}^{72}\exp\left(\kappa \cos\left(\theta_{j}-\theta_{gt}\right)\right)},$$

where $\theta_{gt}$ denotes the ground truth gaze direction, and $\theta_{i}$ denotes the representative angle of the i-th bin. The cosine term measures the angular proximity between two directions in the circular space. The concentration parameter κ controls label sharpness, with larger values approaching hard classification. The held-out validation analysis in Section 4.2 selects κ=6 as the operating point.

#### 3.1.3. Distribution Activation

We adopt the softmax function to obtain the predicted probability distribution, which is formulated as:

(5) $$\hat{p}i=\frac{\exp\left(z_{i}\right)}{\sum_{j=1}^{72}\exp\left(z_{j}\right)},$$

where $z_{i}$ denotes the logit corresponding to the i-th directional bin. In many cases, a softmax temperature parameter T is introduced to control the sharpness of the output distribution, yielding:

(6) $$\hat{p}i=\frac{\exp\left(z_{i}/T\right)}{\sum_{j=1}^{72}\exp\left(z_{j}/T\right)}.$$

When T is small, the output distribution tends to approach hard classification, whereas a larger T produces a smoother distribution.

Softmax converts the direction logits into a normalized circular distribution. Although temperature scaling can modify output sharpness, label dispersion is already controlled by κ. We therefore set T=1 to avoid a second coupled sharpness parameter; uncertainty reliability is examined separately from temperature tuning.

#### 3.1.4. Circular Direction Distribution Learning Network Architecture

CDDL receives the resized head crop and normalized head center. A ResNet-50-CBAM head encoder and a lightweight multilayer perceptron encode appearance and position, respectively; their features are concatenated and mapped to 72 direction logits.

Given an input image, the head region of the target person is first cropped and resized as the head image $I_{h}$. The normalized head position is denoted as $p_{h}=\left(x_{h},y_{h}\right)$, where $x_{h}$ and $y_{h}$ represent the horizontal and vertical coordinates of the head center in the image coordinate system. The proposed direction network consists of three main components: a head appearance encoder, a head position encoder, and a feature fusion classifier.

For the head appearance encoder, we adopt a ResNet-50 backbone enhanced with a convolutional block attention module (CBAM) [54,55]. The head image $I_{h}$ is fed into the backbone to extract a high-level appearance feature. The output feature is then projected by a fully connected transformation to obtain a compact head representation:

(7) $$f_{h}=\phi_{h}\left(I_{h}\right),$$

where $\phi_{h}\left(\cdot \right)$ denotes the head appearance encoder and $f_{h}\in {\mathbb{R}}^{512}$ is the extracted head feature. CBAM is inserted only after the 3 × 3 convolution in layer3. This lightweight modification is inherited from the direction backbone and is not the focus of the present contribution.

For the head position encoder, the normalized head coordinate $p_{h}$ is passed through a lightweight multilayer perceptron to obtain a spatial position embedding:

(8) $$f_{p}=\phi_{p}\left(p_{h}\right),$$

where $\phi_{p}\left(\cdot \right)$ denotes the position encoder and $f_{p}\in {\mathbb{R}}^{256}$ is the encoded position feature. The position embedding supplies global spatial context for the head-dependent direction estimate.

The head appearance feature and the position feature are concatenated and further processed by a fusion module:

(9) $$f=Concat\left(f_{h},f_{p}\right),$$

(10) $$z=\phi_{f}\left(f\right),$$

where $f\in {\mathbb{R}}^{768}$ denotes the fused feature, $\phi_{f}\left(\cdot \right)$ is the fusion classifier, and $z\in {\mathbb{R}}^{N}$ denotes the predicted direction logits. In this work, the full circular direction space is divided into N = 72 discrete direction bins, corresponding to an angular resolution of 5°. Therefore, the network outputs a 72-dimensional logit vector rather than a two-dimensional deterministic direction vector.

The predicted logits are converted into a circular direction probability distribution by the softmax function:

(11) $$\hat{p}i=\frac{\exp\left(z_{i}\right)}{\sum_{j=1}^{N}\exp\left(z_{j}\right)},\;i=1,2,\ldots ,N,$$

where ${\hat{p}}_{i}$ represents the predicted probability of the i-th direction bin.

During training, the predicted circular distribution is supervised by the von Mises-based soft direction label described in the previous subsection. The direction learning objective is defined as the cross-entropy between the predicted distribution and the circular soft label:

(12) $$L_{dir}=-\sum_{i=1}^{N}p_{i}\log{\hat{p}}_{i},$$

where $p_{i}$ denotes the soft label probability of the i-th direction bin. The resulting distribution retains both the most likely direction and neighboring alternatives. It is trained with the von Mises soft label using Equation (12) and subsequently passed to PGGM.

### 3.2. Probabilistic Gaze Geometry Modeling (PGGM)

PGGM converts the CDDL direction distribution into spatial priors without first collapsing it to a single vector. It comprises direction probability aggregation, probability-weighted field generation, and channel-wise volume construction.

#### 3.2.1. Direction Probability Aggregation

The 72-bin distribution is aggregated into 18 non-overlapping groups by summing four adjacent bins, as defined in Equations (14) and (15). This reduces the number of spatial channels and local redundancy while preserving total probability mass. Section 4.3.1 compares 9, 18, and 36 groups under the same protocol.

Specifically, the 72 directional categories are uniformly divided into 18 directional groups, with each group consisting of four consecutive categories and corresponding to an angular range of 20°. Let the directional probability distribution predicted by CDDL be denoted as:

(13) $$P=p_{1},p_{2},\ldots ,p_{72},$$

where $\sum_{i=1}^{72}p_{i}=1$. The aggregated probability of the j-th directional group is defined as:

(14) $${\hat{p}}_{j}=p_{4j}-3+p_{4j-2}+p_{4j-1}+p_{4j},$$

where j=1,2,…,18. The aggregated directional probability distribution is then obtained as:

(15) $$\hat{P}={\hat{p}}_{1},{\hat{p}}_{2},\ldots ,{\hat{p}}_{18},$$

which also satisfies $\sum_{j=1}^{18}{\hat{p}}_{j}=1$.

Aggregation smooths local fluctuations but does not select a single direction; the grouped representation remains probabilistic.

#### 3.2.2. Probabilistic Direction Field Generation

Rather than constructing one field from the maximum probability direction, PGGM generates one field for each direction group and weights it by the corresponding group probability. We examine the Gaussian Strip Field (GSF), Radial Decay Gaussian Field (RDGF), and Cone-like Gaussian Field (CGF) as alternative spatial formulations.

(1) Gaussian Strip Field

To map directional probabilities into the spatial domain, we first construct a Gaussian Strip Field. For the j-th direction group, its central direction is denoted as:

(16) $$\theta_{j}=20{}^{\circ}\times j-10{}^{\circ},$$

where j=1,2,…,18.

The corresponding unit direction vector is defined as:

(17) $$d_{j}=\left[\cos\theta_{j},\sin\theta_{j}\right].$$

For an arbitrary pixel location $\left(x,y\right)$ in the image, the head center h is taken as the origin of the direction field. A directional ray extending from h along the unit direction vector $d_{j}$ can then be constructed. Subsequently, the perpendicular distance from the pixel location to this directional ray is computed as:

(18) $$D_{⊥}\left(x,y,d_{j}\right).$$

To transform directional consistency into a continuous probabilistic response, we adopt a Gaussian kernel to construct the directional response field:

(19) $$F_{j}\left(x,y\right)=\exp\left(-\frac{D_{⊥}{\left(x,y,d_{j}\right)}^{2}}{2\sigma^{2}}\right),$$

where σ denotes the diffusion coefficient of the direction field, which controls the width of the directional response field.

According to this definition, a pixel location receives a high response when it lies close to the directional ray. As the distance from the ray increases, the response value decays continuously in a Gaussian manner.

Since this direction field extends along the directional ray, we refer to it as the Gaussian Strip Field. The proposed field effectively preserves the geometric relationship between gaze directions while maintaining desirable continuity and differentiability.

(2) Radial Decay Gaussian Field

Although the Gaussian Strip Field can characterize directional consistency, its response extends infinitely along the directional ray. As a result, all distant regions are treated as equally reliable, which may introduce invalid responses.

To address this issue, we introduce a radial decay mechanism and construct a Radial Decay Gaussian Field to reflect the property that spatial influence gradually weakens as the distance from the head increases.

We define the projection distance of a pixel location $\left(x,y\right)$ along the direction vector $d_{j}$ as:

(20) $$D_{\gamma }\left(x,y,d_{j}\right),$$

which represents the radial distance from the head center to the pixel location along the directional ray.

The radial decay term is then formulated as:

(21) $$R_{j}\left(x,y\right)=\exp\left(-\frac{D_{\gamma }{\left(x,y,d_{j}\right)}^{2}}{2\tau^{2}}\right),$$

where τ denotes the radial decay scale parameter.

The final direction field is defined as:

(22) $$F_{j}\left(x,y\right)=\exp\left(-\frac{D_{⊥}{\left(x,y,d_{j}\right)}^{2}}{2\sigma^{2}}\right)\times R_{j}\left(x,y\right).$$

This design jointly considers directional consistency and spatial distance constraints. Compared with the Gaussian Strip Field, the Radial Decay Gaussian Field can effectively suppress responses in distant regions, thereby making the spatial prior more concentrated around the head region.

(3) Cone-like Gaussian Field

Conventional gaze-following methods commonly adopt a cone field to represent the gaze direction prior, where the width of the directional field gradually expands as the distance from the head increases. However, the responses of the Radial Decay Gaussian Field tend to shrink substantially toward the neighborhood of the head, which may weaken the localization capability for distant gaze targets. To preserve both probabilistic modeling ability and the structural property of the cone field, we further propose the Cone-like Gaussian Field. CGF retains directional constraints while allowing uncertainty to expand with distance, which is more consistent with the nature of gaze prediction.

Specifically, instead of using a fixed directional field width, we allow the Gaussian standard deviation to dynamically vary with the radial distance:

(23) $$\sigma_{\gamma }(x,y,d_{j})=\sigma_{0}+kD_{\gamma }(x,y,d_{j}),$$

where $\sigma_{0}$ denotes the cone base width near the head and $\sigma_{\gamma }$ denotes the effective standard deviation at each image location. The coefficient k=0.15 controls the linear increase in field width with radial distance. Consequently, CGF remains relatively concentrated near the head and progressively broadens along the forward gaze ray. The cone base width is selected from the held-out validation subset through the sensitivity analysis in Section 4.3.2. The Gaussian response field is defined as:

(24) $$F_{j}\left(x,y\right)=\exp\left(-\frac{D⊥{\left(x,y,dj\right)}^{2}}{2\sigma_{\gamma }^{2}}\right).$$

As the distance increases, the width of the directional field gradually expands, thereby forming a spatial structure similar to a cone field.

Compared with the Gaussian Strip Field and the Radial Decay Gaussian Field, the Cone-like Gaussian Field can better simulate the propagation pattern of spatial uncertainty in the real gaze-following process and preserve a more reasonable search range in distant regions. The held-out validation analysis in Section 4.3.2 selects a base width of 10 pixels, while k remains fixed.

**Figure 2:**
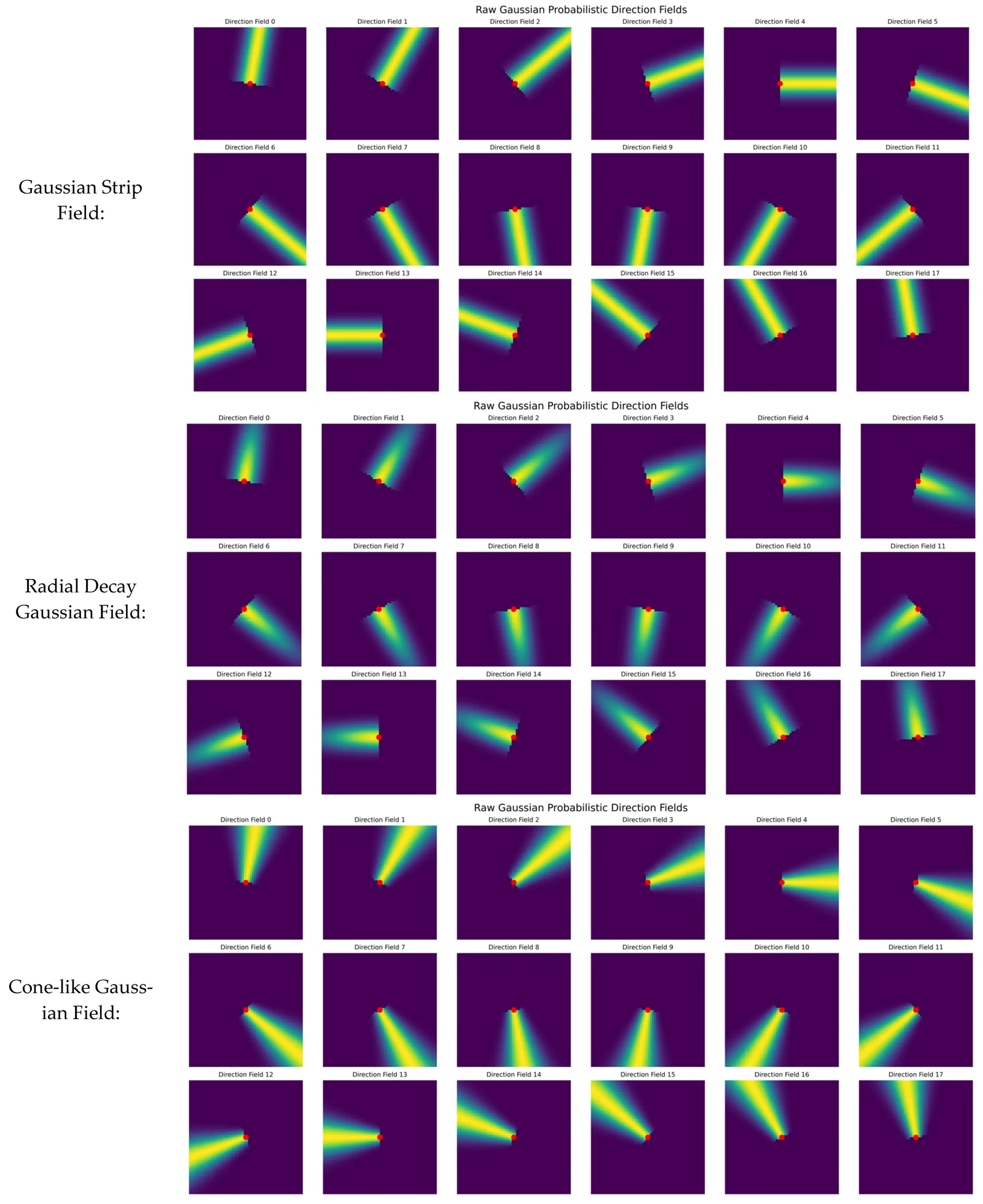
Schematic illustration of the 18 direction groups in the three Probabilistic Direction Field formulations—Gaussian Strip Field (GSF), Radial Decay Gaussian Field (RDGF), and Cone-like Gaussian Field (CGF).

Figure 2 illustrates the schematic diagrams of the three directional fields, each of which contains 18 direction groups. It can be observed that all three proposed probabilistic direction field generation methods are based on Gaussian probabilistic direction fields. Unlike conventional cone fields with hard boundary constraints, Gaussian probabilistic direction fields offer the following advantages:

- They can continuously describe the geometric relationship between spatial locations and directional hypotheses.
- They avoid abrupt information changes caused by hard boundaries.
- They exhibit better robustness and differentiability.

Furthermore, to introduce directional prediction probabilities into the spatial representation, we weight each directional response field using the corresponding direction group probability:

(25) $${\tilde{F}}_{j}\left(x,y\right)={\hat{p}}_{j}\cdot F_{j}\left(x,y\right),$$

where ${\hat{p}}_{j}$ denotes the probability of the j-th direction group, and ${\tilde{F}}_{j}\left(x,y\right)$ denotes the final weighted probabilistic direction field.

Multiplying each field by its group probability embeds the direction distribution into the spatial response: high-probability groups produce stronger fields, while lower probability alternatives remain available.

Finally, 18 probabilistic direction fields are obtained:

(26) $$\left\{{\tilde{F}}_{1},{\tilde{F}}_{2},\ldots ,{\tilde{F}}_{18}\right\}.$$

These probabilistic direction fields are further used in the next section to construct the Direction Probability Volume, which serves as an important geometric prior input for the heatmap network.

#### 3.2.3. Direction Probability Volume Construction

After Direction Probability Aggregation and Probabilistic Direction Field Generation, 18 probabilistic direction fields can be obtained:

(27) $$\tilde{F}=\left\{{\tilde{F}}_{1},{\tilde{F}}_{2},\ldots ,{\tilde{F}}_{18}\right\},$$

where each probabilistic direction field corresponds to the spatial probability response distribution of one direction group.

An intuitive strategy is to fuse all probabilistic direction fields by summation to obtain a single direction field representation. However, this operation may cause information entanglement among different directional hypotheses, making it difficult for the network to distinguish the independent geometric constraints associated with different direction groups.

The probability-weighted fields are stacked rather than summed. Channel-wise stacking preserves the source direction of each response and prevents distinct hypotheses from being merged before scene-based target localization.

Specifically, the 18 probabilistic direction fields are concatenated along the channel dimension:

(28) $$V=Concat\left({\tilde{F}}_{1},{\tilde{F}}_{2},\ldots ,{\tilde{F}}_{18}\right),$$

where $V\in {\mathbb{R}}^{18\times H\times W}$, H and W denote the spatial resolution of the direction fields, respectively.

Each channel is jointly determined by group probability and field geometry. The resulting Direction Probability Volume therefore retains the grouped direction distribution in spatial form and allows the Heatmap Prediction Network to combine distinct direction-to-region hypotheses with scene evidence.

Furthermore, the Direction Probability Volume is fused with the original RGB image at the channel level:

(29) $$I_{input}=Concat\left(I_{RGB},V\right),$$

where $I_{RGB}\in {\mathbb{R}}^{3\times H\times W}$ denotes the input scene image. The final input to the Heatmap Prediction Network is therefore obtained as:

(30) $$X\in {\mathbb{R}}^{21\times H\times W},$$

where 21 = 3 + 18, corresponding to 3 RGB channels and 18 probabilistic direction field channels.

The 21-channel input combines RGB appearance with 18 direction field channels. The Heatmap Prediction Network can therefore resolve multiple geometric hypotheses using scene evidence rather than relying on one deterministic direction.

#### 3.2.4. Design Analysis of the Direction Probability Volume

The Direction Probability Volume is constructed by stacking probability-weighted Probabilistic Direction Fields along the channel dimension. The number of direction groups controls a trade-off between angular resolution, representation size, and redundancy among neighboring direction bins. We therefore compare 9, 18, and 36 groups under a matched protocol in Section 4.3.1. The 18-group configuration is retained as an empirically supported operating point. Channel-wise stacking preserves the identity of the direction groups so that the Heatmap Prediction Network can combine distinct direction-to-region hypotheses with scene appearance during target localization.

### 3.3. Heatmap Network Construction

Image-level early fusion concatenates the RGB image and Direction Probability Volume before feature extraction, as defined in Equation (31). This allows the backbone to learn appearance and geometry jointly while preserving the direction group channels.

Let $I_{RGB}\in {\mathbb{R}}^{3\times H\times W}$ denote the input RGB image, and let $V\in {\mathbb{R}}^{K\times H\times W}$ denote the PGGM volume generated from the learned circular direction distribution, where K is the number of aggregated direction groups. Each channel in V corresponds to a spatial probabilistic gaze map under a specific direction hypothesis, and its response is weighted by the predicted direction probability. Instead of selecting only the most confident directions, we preserve the complete PGGM volume because the full direction distribution contains useful uncertainty information for gaze target localization. The input to the heatmap network is therefore constructed as:

(31) $$X=Concat\left(I_{RGB},V\right)\in {\mathbb{R}}^{\left(3+K\right)\times H\times W}.$$

For the selected 18-group configuration, the network receives a 21-channel input. The comparison with feature-level fusion is reported in Section 4.3.3.

The heatmap prediction module is built upon a ResNet-50 backbone with a Feature Pyramid Network (FPN) [54,56]. Since the standard ImageNet-pretrained ResNet-50 accepts a 3-channel RGB image, we modify the first convolutional layer to accept the 21-channel early-fusion input. Specifically, the original RGB convolutional kernels are retained for the first three channels, while the kernels of the additional PGGM channels are initialized by the channel-wise mean of the pretrained RGB kernels. This initialization strategy preserves the pretrained visual representation while allowing the network to adapt to the newly introduced probabilistic geometry maps.

The modified ResNet-50 extracts hierarchical visual-geometric features from the fused input. The FPN then aggregates multi-level features and produces a high-resolution feature representation for heatmap prediction. Finally, a lightweight prediction head composed of convolutional layers maps the fused feature representation into a single-channel heatmap logit map:

(32) $$\hat{Y}=H_{\theta }\left(X\right),$$

where $H_{\theta }\left(\cdot \right)$ denotes the heatmap network and $\hat{Y}\in {\mathbb{R}}^{1\times h\times w}$ is the predicted heatmap logit. In this work, the output resolution is set to 56×56. During training, $\hat{Y}$ is supervised by a Gaussian heatmap generated from the ground truth gaze point using binary cross-entropy with logits. During inference, a sigmoid function is applied to obtain the gaze probability heatmap, and the location with the maximum response is selected as the predicted gaze point.

The predicted logit map is supervised by a Gaussian target heatmap during training; at inference, sigmoid activation is applied, and the maximum response defines the predicted gaze point. This construction links the direction distribution to scene-conditioned target localization without introducing a separate semantic detector.

### 3.4. Loss Function

For the Circular Direction Distribution Learning Network, we define a Circular Direction Distribution Loss. Since both the ground truth label and the network prediction are probability distributions, we adopt soft cross-entropy for stable optimization. The loss is formulated as:

(33) $$L=-\sum_{i}p_{i}\log{\hat{p}}_{i},$$

where $p_{i}$ denotes the ground truth probability of the i-th directional bin, and ${\hat{p}}_{i}$ denotes the corresponding predicted probability. The loss used to train CDDL is therefore defined as:

(34) $$L_{dir}=-\sum_{i=1}^{72}p_{i}\log{\hat{p}}_{i}.$$

This objective directly matches the soft direction label and requires no additional normalization.

For the Heatmap Prediction Network, we define a Heatmap Regression Loss. Specifically, we adopt the binary cross-entropy heatmap loss, which is given by:

(35) $$L_{heat}=-\frac{1}{HW}\sum_{x=1}^{W}\sum_{y=1}^{H}\left[G\left(x,y\right)\log\hat{G}\left(x,y\right)+\left(1-G\left(x,y\right)\right)\log\left(1-\hat{G}\left(x,y\right)\right)\right],$$

where $G\left(x,y\right)$ and $\hat{G}\left(x,y\right)=\mathrm{Sigmoid}(\hat{Y})$ denote the ground truth heatmap and the predicted heatmap at spatial location $\left(x,y\right)$, respectively.

The framework is trained in two sequential stages. In Stage 1, CDDL is optimized using the direction-distribution loss defined in Equation (34). In Stage 2, the trained CDDL is fixed, and the Heatmap Prediction Network is optimized using the heatmap regression loss defined in Equation (35), with the Direction Probability Volume generated by CDDL. The corresponding stage-specific objectives are:

(36) $$L^{\left(1\right)}=L_{dir},\;\;\;\;L^{\left(2\right)}=L_{heat}.$$

Accordingly, the two stages are optimized with separate objectives rather than a weighted joint loss.

## 4. Experiments

### 4.1. Experimental Setup

#### 4.1.1. Dataset

We evaluate the proposed method on the GazeFollow dataset [9], which is a large-scale benchmark dataset designed for general gaze following in natural images. The goal of gaze following is to predict the spatial location that a target person is looking at, given an image and the head location of that person. Compared with conventional saliency prediction, gaze following is more person-centered, since the target location depends not only on the visual content of the image but also on the head position, head orientation, and possible line of sight of the selected person.

GazeFollow was constructed from several large-scale image datasets containing people, including SUN, MS COCO, Actions 40, PASCAL, ImageNet Detection Challenge, and Places. Specifically, the dataset was collected from 1548 images from SUN, 33,790 images from MS COCO, 9135 images from Actions 40, 7791 images from PASCAL, 508 images from the ImageNet Detection Challenge, and 198,097 images from Places. Since these source datasets do not provide gaze annotations, the authors annotated gaze locations using Amazon Mechanical Turk. Annotators were asked to mark the center of the target person’s eyes and the location where the person was believed to be looking. They could also indicate cases where the person was looking outside the image or where the head was not visible. After quality control, the dataset contains 130,339 people in 122,143 images with gaze locations inside the image.

Following the standard setting of GazeFollow, the dataset is divided into disjoint training and testing subsets. The test split contains approximately 4782 people, while the remaining samples are used for training. To avoid data leakage, all people from the same image are assigned to the same split. In addition, the test images were selected such that the fixation locations are approximately uniformly distributed across the image. For the test set, 10 gaze annotations were collected for each target person to evaluate human consistency and the multimodal nature of gaze-following annotations. This is important because different human observers may provide different but still plausible gaze targets, especially in ambiguous scenes or scenes containing multiple salient objects.

In our experiments, we follow the common evaluation setting of GazeFollow and assume that the head/eye position of the target person is available during both training and testing. For each training sample, the mean gaze location is used to generate the Gaussian ground truth heatmap for heatmap supervision.

For cross-dataset evaluation, we additionally use the VideoAttentionTarget (VAT) dataset [51]. VAT contains video frames annotated with person locations, gaze targets, and in-/out-of-frame gaze status. The official VAT test split contains 31,978 person frames, of which 20,655 have in-frame gaze targets. Because the present framework predicts an in-frame heatmap and does not include an out-of-frame classification branch, the cross-dataset analysis is restricted to these 20,655 in-frame person frames. All evaluated models were trained exclusively on GazeFollow and were applied to VAT without fine-tuning, model selection, or configuration adjustment.

#### 4.1.2. Evaluation Metrics

Evaluation was conducted at two levels: the gaze direction distribution predicted by the Circular Direction Distribution Learning (CDDL) network and the final gaze target location predicted by the Heatmap Prediction Network. All test-set metrics were computed using every valid human annotation available for each sample; no fixed upper limit was imposed on the number of annotations.

**Direction stage metrics.** The CDDL network predicts a circular probability distribution over B=72 direction bins. Let ${\hat{d}}_{i}$ denote the predicted unit direction for sample i, obtained from the center angle of the maximum-probability bin. Let $e_{i}$ be the annotated eye position, and let $G_{i}=\{g_{ij}{\}}_{j=1}^{N_{i}}$ contain the $N_{i}$ valid gaze annotations. Their mean position is:

(37) $$\mu_{i}=\frac{1}{N_{i}}\sum_{j=1}^{N_{i}}g_{ij}.$$

The mean annotation reference direction and the direction from the eye to the annotation j are denoted by:

(38) $$d_{i}^{\mu }=\frac{\mu_{i}-e_{i}}{∥\mu_{i}-e_{i}{∥}_{2}},\;\;d_{ij}=\frac{g_{ij}-e_{i}}{∥g_{ij}-e_{i}{∥}_{2}}.$$

Direction Angular Error (Dir-Ang) measures the angular difference between ${\hat{d}}_{i}$ and $d_{i}^{\mu }$, whereas Direction Minimum Angular Error (Dir-MAng) independently selects the smallest angular error over the valid annotation directions:

(39) $${Dir-Ang}_{i}=arccos\;\left(clip\;\left({\hat{d}}_{i}^{T}d_{i}^{\mu },-1,1\right)\right),$$

(40) $${Dir-MAng}_{i}={min}_{j}arccos\;\left(clip\;\left({\hat{d}}_{i}^{T}d_{ij},-1,1\right)\right).$$

Any annotation for which $g_{ij}=e_{i}$ is excluded from Dir-MAng because its direction is undefined. In addition, Top-1, Top-3, and Top-5 Accuracy indicate whether the discretized mean annotation direction is included among the k∈{1,3,5} bins assigned the highest predicted probabilities. Dir-Ang is used as the primary direction stage localization metric, while the Top-k measures characterize the ranking of plausible directional hypotheses.

**Final gaze target metrics.** Let $H_{i}\left(x\right)$ be the predicted gaze heatmap, and let:

(41) $${\hat{g}}_{i}=\underset{x}{arg\;max}\;H_{i}\left(x\right)$$

be its peak location in normalized image coordinates. We report two AUC protocols because prior GazeFollow studies do not use a single interchangeable implementation. The primary AUC used in the historical comparison is the conventional 5×5 Pooled/Global AUC. For each sample, the predicted heatmap is resized to 5×5, all valid annotations are mapped to a binary 5×5 target map, and the flattened predictions and targets are concatenated across the complete test set before the receiver operating characteristic AUC is calculated. This protocol is used for direct comparison with the direction field baseline of Lian et al.

An Original Resolution Per-Image Point AUC is additionally reported for the controlled model comparisons and supplementary analyses. Under this protocol, the prediction is resized to the original image resolution, all valid annotations are represented as positive points, AUC is computed separately for each sample, and the resulting values are averaged. Because the two AUC protocols differ in both spatial resolution and cross-sample aggregation, their numerical values are not treated as directly interchangeable.

Localization errors are calculated in normalized $\left[0,1\right]\times \left[0,1\right]$ image coordinates. Dist is the Euclidean distance between the predicted peak and the mean annotation, whereas MDist is the minimum Euclidean distance to any valid annotation:

(42) $${Dist}_{i}=∥{\hat{g}}_{i}-\mu_{i}{∥}_{2},\;\;{MDist}_{i}={min}_{j}∥{\hat{g}}_{i}-g_{ij}{∥}_{2}.$$

For angular evaluation, define the predicted gaze vector, mean annotation gaze vector, and individual annotation vectors as ${\hat{v}}_{i}={\hat{g}}_{i}-e_{i}$, $v_{i}^{\mu }=\mu_{i}-e_{i}$, and $v_{ij}=g_{ij}-e_{i}$, respectively. Ang is computed with respect to the mean annotation, while MAng independently selects the smallest valid annotation-wise angular error:

(43) $${Ang}_{i}=arccos\;\left(clip\;\left(\frac{{\hat{v}}_{i}^{T}v_{i}^{\mu }}{∥{\hat{v}}_{i}{∥}_{2}∥v_{i}^{\mu }{∥}_{2}},-1,1\right)\right),$$

(44) $${MAng}_{i}={min}_{j}arccos\;\left(clip\;\left(\frac{{\hat{v}}_{i}^{T}v_{ij}}{∥{\hat{v}}_{i}{∥}_{2}∥v_{ij}{∥}_{2}},-1,1\right)\right).$$

The inverse–cosine input is clipped to $\left[-1,1\right]$ for numerical stability, and angular errors are reported in degrees. Any zero-length annotation vector is excluded from the corresponding angular minimum. MDist and MAng are minimized independently; therefore, the annotations attaining the two minima need not be identical. Dataset-level Dir-Ang, Dir-MAng, Dist, MDist, Ang, and MAng are obtained by averaging their valid sample-level values. Higher values indicate better performance for AUC and Top-k Accuracy, whereas lower values are better for all distance and angular error metrics.

**Validation-based model selection quantities.** The held-out validation subset was derived from the official training split and contains one gaze annotation per person instance. Consequently, MDist and MAng are redundant with Dist and Ang on this subset. Section 4.3 therefore report only the predefined heatmap-selection quantities: per-image AUC at the prediction heatmap resolution, Dist, and mean binary cross-entropy loss. These quantities are used for model selection and are distinct from the final test set metrics defined above.

#### 4.1.3. Training Strategy

We adopted a two-stage training protocol that separates gaze direction distribution learning from gaze target localization. A held-out validation subset was derived from the official GazeFollow training split. To prevent image-level leakage, annotations were grouped by image identity before partitioning, yielding 112,988 training instances and 12,569 validation instances; person instances from the same image were assigned to the same subset. The official GazeFollow test set was reserved exclusively for final evaluation and was not used for optimization, learning rate scheduling, hyperparameter selection, model selection, or early stopping.

In the first stage, the Circular Direction Distribution Learning (CDDL) network was optimized on the training subset. The concentration parameter and learning rate schedule were selected according to validation performance. The selected configuration used κ=6 with StepLR, and the model selected at epoch 6 was used to generate the direction representations for all subsequent experiments.

In the second stage, the selected CDDL network was kept fixed while the Heatmap Prediction Network was trained. The parameters and normalization statistics of the direction branch were not updated during this stage. All heatmap variants used the same training and validation subsets, backbone initialization, optimizer, learning rate schedule, batch size, random seed, and model selection criterion. Each variant was trained for seven epochs. Selection was based primarily on per-image AUC computed at the prediction heatmap resolution; exact ties were resolved using lower Dist and then lower mean binary cross-entropy loss. After all model and hyperparameter choices had been completed on the validation subset, the selected models were evaluated on the official test set using the protocol described in Section 4.1.2.

#### 4.1.4. Implementation Details

The implementation was developed in Python 3.11.14 using PyTorch 2.12.0, CUDA 13.0.1, and cuDNN 9.2.0. Training and evaluation were conducted on a workstation equipped with an Intel Core i7-12700KF processor, 64 GB of system memory, and one NVIDIA GeForce RTX 5090 D v2 GPU with 24 GB of video memory. The complete hardware and software configuration is summarized in Table 1.

Computational efficiency was measured in FP32 on the same GPU using forward-only inference with fixed 224 × 224 input tensors. Batch size 1 was used to assess latency, and batch size 64 was used to assess throughput and peak GPU memory. Each configuration was measured over five repetitions after dedicated warm-up iterations; data loading, host-to-device transfer, metric computation, and CPU post-processing were excluded. Full benchmarking details are provided in Supplementary Methods S3.

The CDDL network used a ResNet-50 backbone with a Convolutional Block Attention Module (CBAM) and ImageNet initialization. It was optimized using Adam with an initial learning rate of $1\times 10^{-4}$ and a weight decay of $5\times 10^{-4}$. For the selected configuration, StepLR reduced the learning rate by a factor of 0.5 every five epochs. The concentration parameter was κ=6.

The Heatmap Prediction Network used the modified ResNet-50 feature-pyramid architecture described in Section 3 and was initialized from ImageNet-pretrained ResNet-50 weights. AdamW was used with an initial learning rate of $1\times 10^{-4}$ and a weight decay of $1\times 10^{-4}$. The learning rate was reduced to $1\times 10^{-5}$ at epoch 6 and to $8\times 10^{-6}$ at epoch 7. Training was performed in FP32 with a batch size of 64. The supervision heatmap was generated at the prediction resolution using a Gaussian kernel with a standard deviation of 3 pixels, and the network was optimized using binary cross-entropy with logits.

The final configuration combined the selected CDDL model (κ = 6) with the complete 18-channel Direction Probability Volume. The probabilities of the 72 direction bins were aggregated into 18 direction groups, and each group was projected using the Cone-like Gaussian Field (CGF). A cone-base width of 10 pixels at the network input resolution was adopted according to the held-out validation analysis. This setting is treated as an operating point within the evaluated range rather than as a theoretically unique optimum. The complete Direction Probability Volume was concatenated with the RGB scene image through image-level early fusion at the input level, producing the 21-channel input defined in Section 3.2.3.

### 4.2. Effectiveness of Circular Direction Distribution Learning

This section evaluates the concentration parameter and learning rate schedule used by CDDL and then compares the resulting circular direction distribution with deterministic direction regression. All rows report the test performance of models selected exclusively on the image-grouped validation split described in Section 4.1.3. The official GazeFollow test set was not used to determine κ, the learning rate schedule, or the selected epoch.

#### 4.2.1. Influence of the Concentration Parameter and Learning Rate Schedule

The concentration parameter κ controls the sharpness of the von Mises supervision distribution. Small values spread probability mass over a wider angular neighborhood, whereas large values approach a more concentrated target. We first evaluated κ∈{2,3,4,5,6,8} under a fixed learning rate and then examined StepLR for the two representative settings κ=4 and κ=6. Table 2 reports the final test performance of the corresponding validation-selected models.

The fixed learning rate sweep shows a non-monotonic relationship between κ and direction accuracy, indicating that neither an overly diffuse nor an overly concentrated soft target is uniformly preferable. The validation sweep selected κ=6 among the fixed learning rate settings. Applying StepLR at this concentration further reduced Dir-Ang by 1.01° and Dir-MAng by 0.91° relative to fixed rate training, while increasing Top-1, Top-3, and Top-5 Accuracy by 1.51, 2.43, and 2.32 percentage points, respectively. These paired improvements remained supported after Holm correction ($p_{Holm}<1.2\times 10^{-5}$ for both angular metrics).

The final κ=6 StepLR model also produced numerically lower Dir-Ang and Dir-MAng than the κ=4 StepLR model. However, the Dir-Ang difference was not statistically supported after Holm correction ($p_{Holm}=0.297$). We therefore regard κ=6 as the validation-selected operating point used in the subsequent experiments, rather than as a theoretically unique optimum.

#### 4.2.2. Comparison with Deterministic Direction Regression

To examine whether the benefit originates from circular distribution learning rather than only from the learning rate schedule, Table 3 includes both a fixed learning rate CDDL model and the final StepLR model. The fixed learning rate comparison isolates the output formulation under a matched schedule, whereas the StepLR row reports the final direction model used by the complete framework.

Under the matched fixed learning rate setting, CDDL reduced Dir-Ang from 22.74° to 19.72° and Dir-MAng from 13.32° to 10.80°. These changes correspond to relative reductions of 13.25% and 18.88%, with bootstrap 95% confidence intervals of $\left[2.41{}^{\circ},3.61{}^{\circ}\right]$ and $\left[2.03{}^{\circ},3.00{}^{\circ}\right]$ for the absolute reductions, respectively. Both paired differences remained significant after Holm correction ($p_{Holm}<4.2\times 10^{-34}$).

The final CDDL configuration further reduced Dir-Ang to 18.72° and Dir-MAng to 9.90°. Relative to deterministic regression, the absolute reductions were 4.02° and 3.42°, equivalent to 17.68% and 25.68%, respectively; the corresponding bootstrap 95% confidence intervals were $\left[3.41{}^{\circ},4.64{}^{\circ}\right]$ and $\left[2.91{}^{\circ},3.93{}^{\circ}\right]$, and both paired tests remained significant after Holm correction ($p_{Holm}<1.6\times 10^{-51}$). The controlled fixed-rate result indicates that the circular distribution formulation itself improves direction estimation, while StepLR provides an additional optimization benefit. These findings support CDDL as a probabilistic intermediate representation for the subsequent direction-to-region projection, without interpreting the predicted distribution as a direct measure of human perceptual uncertainty.

### 4.3. Effectiveness of Probabilistic Gaze Geometry Modeling

The ablations in this section determine the Probabilistic Gaze Geometry Modeling configuration using the held-out validation subset derived from the official training split. Table 4, Table 5 and Table 6 therefore report model selection analyses rather than final benchmark comparisons. AUC denotes per-image AUC at the prediction heatmap resolution, Dist denotes peak localization distance, and BCE denotes mean binary cross-entropy loss. Because each validation instance contains one gaze annotation, MDist and MAng are identical to Dist and Ang, respectively. Ang is not included because it was not part of the predefined heatmap model selection criterion. Complete test set results for the principal controlled variants are provided in Supplementary Table S1.

#### 4.3.1. Probabilistic Direction Field Formulation and Direction Group Count

We first compared Gaussian Strip Field (GSF), Cone-like Gaussian Field (CGF), and Radial Decay Gaussian Field (RDGF) using the same complete 18-channel Direction Probability Volume. GSF applies a fixed transverse width along the gaze direction, CGF expands its spatial tolerance with distance from the head, and RDGF additionally attenuates responses along the radial axis. Table 4a shows that GSF achieved the highest AUC and the lowest BCE on the held-out validation subset, whereas CGF obtained the lowest Dist. RDGF yielded a lower AUC and a larger Dist than the other two variants.

These results indicate a modest trade-off between heatmap discrimination and peak localization rather than a uniform ranking of the three formulations. CGF is retained because its distance-dependent expansion directly implements the intended propagation from directional uncertainty to image-space tolerance, not because it dominates every validation criterion.

Table 4b evaluates the aggregation of the 72 direction bins into 9, 18, or 36 direction groups before spatial projection. The 18-group representation achieved the highest AUC by a small margin and the lowest Dist and BCE. The 36-group representation produced a similar AUC but a larger Dist and twice as many direction field channels. We therefore retained 18 groups as a practical balance between angular resolution, localization quality, and representation size.

#### 4.3.2. Cone Base Width Sensitivity

To examine the sensitivity of CGF to its initial transverse spread, the cone base width $\sigma_{0}$ was evaluated at 2, 4, 6, 8, and 10 pixels while k=0.15 and all other training settings were held constant. As shown in Table 5, performance varied only modestly across the evaluated range. A base width of 10 pixels achieved the highest AUC and the lowest BCE, whereas 4 pixels yielded the lowest Dist. Following the predefined validation criterion, 10 pixels was retained as the operating point, while the small metric trade-off is reported explicitly.

#### 4.3.3. Fusion Strategy

We next compared feature-level fusion with two image-level early-fusion variants. Feature-level Full-18 concatenates the Direction Probability Volume with the feature pyramid representation. In contrast, image-level early fusion concatenates the Direction Probability Volume with the RGB scene image before visual feature extraction. For concision, Full-18 denotes the complete 18-channel Direction Probability Volume, whereas Top-3 retains only the three channels associated with the highest aggregated direction group probabilities.

Table 6 shows that both image-level variants obtained substantially lower Dist and BCE than feature-level Full-18 on the held-out validation subset. Image-level Top-3 achieved the highest AUC and the lowest BCE, whereas image-level Full-18 yielded a marginally lower Dist. The final framework retains image-level Full-18 because it preserves all direction group channels and the complete direction-to-region representation; the validation results do not establish a uniform performance advantage over Top-3.

### 4.4. Protocol-Aware Comparison with Reference Methods

A direct ranking of all published GazeFollow results is complicated by differences in heatmap resolution, cross-sample aggregation, task formulation, input cues, and pretraining resources. Table 7 therefore organizes the comparison by evaluation compatibility.

To preserve continuity with the original GazeFollow benchmark and with the comparison reported by Lian et al., the table first retains the non-learned baselines, saliency baselines, original Recasens model, the ResNet-50 reimplementation reported by Lian et al., and the One Human reference. Direct pooled/global comparison is then restricted to Lian et al. and the present study, whereas recent per-image or per-person-instance evaluators and methods using additional cues or training resources are reported separately.

Within Table 7a, the proposed model achieves a pooled/global AUC of 0.9066, slightly exceeding the 0.9060 reported by Lian et al., and obtains lower Dist, MDist, Ang, and MAng. The numerical advantages in AUC, Dist, and MDist are small, whereas the angular differences are more pronounced: Ang is reduced by 0.70° and MAng by 0.57°, corresponding to relative reductions of approximately 4.0% and 6.5%, respectively. This pattern is consistent with the design of CDDL, which directly supervises a circular distribution in direction space, and with PGGM, which transfers the retained directional hypotheses into spatial priors for target localization. Because this comparison is cross-paper, it does not by itself establish causality; the mechanistic interpretation is instead supported by the matched Direction Regression–CDDL and deterministic–probabilistic comparisons in Section 4.2 and Section 4.3.

Table 7b places the method within the contemporary evaluation landscape. Recent Transformer and foundation–encoder approaches report higher AUC values under modern per-image or per-person-instance evaluators. The proposed model uses a conventional ResNet-50-based direction and heatmap architecture and does not rely on a foundation-scale visual encoder, multi-person Transformer reasoning, additional modalities, or external gaze-pretraining datasets. Its original resolution per-image AUC of 0.9214 therefore shows that an explicit, task-specific direction-to-region representation can remain competitive with conventional scene–head models, although a clear gap remains between the proposed model and recent large-scale or relation-intensive architectures. Future work will investigate whether stronger visual encoders and relational reasoning can improve target commitment while retaining the interpretability of CDDL–PGGM.

Table 7c further shows that depth, three-dimensional geometry, pose, local/global coordination, and additional gaze-pretraining provide complementary information for target disambiguation. These cues are not alternatives to direction space uncertainty modeling; they address the remaining ambiguity after a plausible direction has been inferred. A natural extension is therefore to combine the explicit CDDL–PGGM prior with stronger visual backbones, object-level semantic relation modeling, and distance-aware target commitment. Such extensions may improve selection among multiple plausible objects while preserving the explicit uncertainty and direction-to-region interpretation of the present framework.

### 4.5. Additional Quantitative Evaluation

We further evaluate zero-shot cross-dataset transfer, computational efficiency, and internal uncertainty reliability to assess the practical scope, uncertainty behavior, and error characteristics of the proposed representation beyond the principal in-domain GazeFollow results.

#### 4.5.1. Zero-Shot Cross-Dataset Evaluation on VideoAttentionTarget

The RGB-only, deterministic CGF, and proposed Full-18 CGF models were trained exclusively on GazeFollow and evaluated directly on the 20,655 in-frame person frames of the official VAT test split. VAT was not used for fine-tuning, model selection, or configuration adjustment. Because the present framework does not include an out-of-frame classification branch, the analysis focuses on in-frame localization and does not report out-of-frame average precision. The detailed evaluation result is shown in Table 8.

Introducing explicit deterministic gaze geometry produces a substantial cross-dataset gain over RGB-only prediction: AUC increases from 0.8170 to 0.8737, while VAT Distance and angular error decrease from 0.2714 to 0.1666 and from 36.30° to 20.33°, respectively. This result indicates that the head-centered direction-to-region prior transfers more reliably than appearance-only prediction to the video-domain imagery of VAT.

Retaining the full probabilistic Direction Probability Volume provides a further, smaller gain over deterministic CGF. AUC increases by 0.0086, short side-normalized distance decreases by 0.0087, and angular error decreases by 0.85°. The mean VAT Distance is also lower by 0.0092, although the paired rank-based evidence for this metric is mixed. The extended paired analysis is reported in Supplementary Table S2. These findings show that the benefit of preserving multiple directional hypotheses is not confined to GazeFollow, but they do not establish uniform cross-domain superiority. The coverage-limited ChildPlay available-subset results in Supplementary Table S3 further illustrate this domain dependence.

#### 4.5.2. Computational Efficiency

As shown in Table 9, we evaluated the computational cost of the Direction Probability Volume using three representative configurations: an RGB-only model, a deterministic CGF model with one direction field channel, and the proposed Full-18 CGF model with the complete 18-channel Direction Probability Volume. All measurements used FP32 forward inference on the same NVIDIA GeForce RTX 5090 D v2 GPU. Batch size 1 was used for latency, whereas batch size 64 was used for throughput and peak GPU memory. Each configuration was measured over five repetitions after dedicated warm-up iterations. Table 9 shows the computational efficiency of representative model configurations. Data loading, host-to-device transfer, metric computation, and CPU post-processing were excluded; the complete protocol and extended results are provided in Supplementary Methods S3 and Table S4.

Relative to deterministic CGF, the full probabilistic representation adds 53,312 inference-active parameters, corresponding to an increase of 0.10%. The larger input volume nevertheless introduces measurable runtime and memory costs: batch-1 latency increases from 9.52 to 12.54 ms per sample, batch-64 throughput decreases from 1313.55 to 1143.48 samples/s, and peak allocated GPU memory increases from 1.52 to 2.19 GB. These changes correspond to a 31.7% latency increase, a 12.9% throughput reduction, and a 44.0% increase in peak allocated memory. Thus, the parameter increase is small, whereas the latency and memory trade-offs are non-trivial and should be considered in resource-constrained applications.

The RGB-only configuration remains substantially lighter because it bypasses both the direction estimation branch and the Direction Probability Volume. Comparison with deterministic CGF therefore isolates the additional cost of retaining multiple probabilistic direction channels, whereas comparison with RGB-only reflects the cost of the complete two-stage direction-guided formulation.

#### 4.5.3. Internal Uncertainty Reliability and Error Characterization

To examine whether predictive spread reflects annotation ambiguity and localization difficulty, we analyzed normalized direction entropy, normalized heatmap entropy, the area containing 90% of the predicted probability mass, annotation dispersion, and localization error across all 4782 GazeFollow test instances. Annotation dispersion shows a weak association with direction entropy (Spearman correlation = 0.146), but clearer associations with heatmap entropy (0.426) and 90% mass area (0.413). Annotation dispersion and heatmap entropy are also associated with Dist (0.499 and 0.498, respectively), whereas the association between direction entropy and Dist is weaker (0.189).

The quantile analysis exhibits a monotonic increase in localization difficulty as predictive uncertainty rises. From the lowest to the highest heatmap entropy quintile, Dist increases from 0.0537 to 0.2393 and Ang from 8.05° to 26.56°; annotation dispersion simultaneously increases from 0.0418 to 0.1078. Across low-, medium-, and high-dispersion strata, the proposed model’s original resolution per-image AUC decreases from 0.9704 to 0.8587, while Dist increases from 0.0872 to 0.2080. Relative to deterministic CGF, the proposed model retains slightly higher AUC and lower Dist and MDist in all three dispersion strata, but its angular advantage is not uniform. We therefore interpret heatmap spread as an informative indicator of internal uncertainty reliability and error risk, rather than as a calibrated estimate of human uncertainty. Full correlation, quantile, dispersion-stratified, and high error analyses are provided in Supplementary Tables S5–S8.

### 4.6. Qualitative Analysis

#### 4.6.1. Direction Distribution Visualization

**Figure 3:**
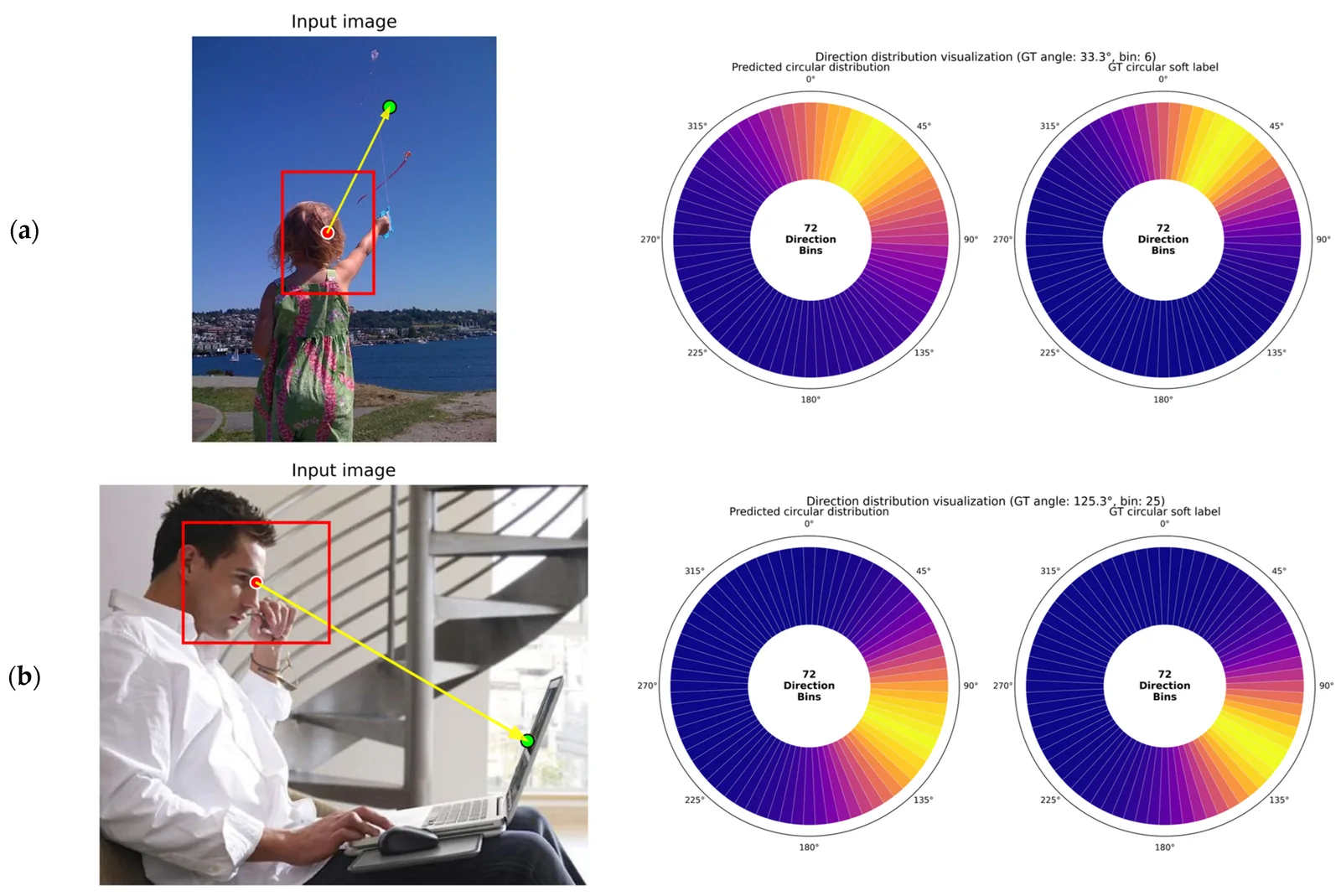
Direction distribution visualization of CDDL. Sample (**a**) shows a challenging back-view case without visible facial cues, while sample (**b**) shows a side-view case with clear head orientation. For each sample, the predicted circular direction distribution is compared with the ground truth circular soft label. The visualization shows that CDDL can preserve directional uncertainty in ambiguous cases while producing more concentrated distributions when reliable head-pose cues are available. The color meanings in the circular distribution are detailed in Figure 1.

To qualitatively analyze whether CDDL can learn meaningful gaze direction distributions, we visualize the predicted circular direction distributions of two representative samples, as shown in Figure 3. The first sample is a challenging back-view case, where the target person’s face and eyes are not visible. The second sample contains a clear side-view face, where the head orientation provides stronger visual evidence for gaze direction estimation. These two cases are selected because they represent two common situations in general gaze following: uncertain gaze inference under weak head cues and relatively reliable gaze inference under visible head pose cues.

Figure 3 compares the predicted circular direction distribution with the ground truth circular soft label. For the back-view sample, the absence of facial and eye cues makes deterministic gaze estimation difficult. In this case, the predicted distribution does not simply collapse into an arbitrary single direction. Instead, it preserves probability responses around plausible neighboring directions, indicating that CDDL can represent directional uncertainty when the visual evidence is weak. This behavior is consistent with the motivation of Circular Direction Distribution Learning: gaze direction should not always be treated as a single deterministic vector, especially when the target person is observed from the back or when the eyes are invisible.

For the side-view sample, the predicted distribution is more concentrated around the ground truth direction, because the visible facial orientation provides stronger cues for gaze estimation. Meanwhile, the model still assigns non-zero probabilities to adjacent direction bins, which is reasonable because gaze annotations in natural images may contain inter-observer variation. This shows that CDDL can maintain a balance between directional discriminability and uncertainty tolerance. Overall, the visualization demonstrates that the proposed direction network learns interpretable circular probability distributions rather than overconfident deterministic gaze directions.

#### 4.6.2. PGGM Probability Volume Visualization

**Figure 4:**
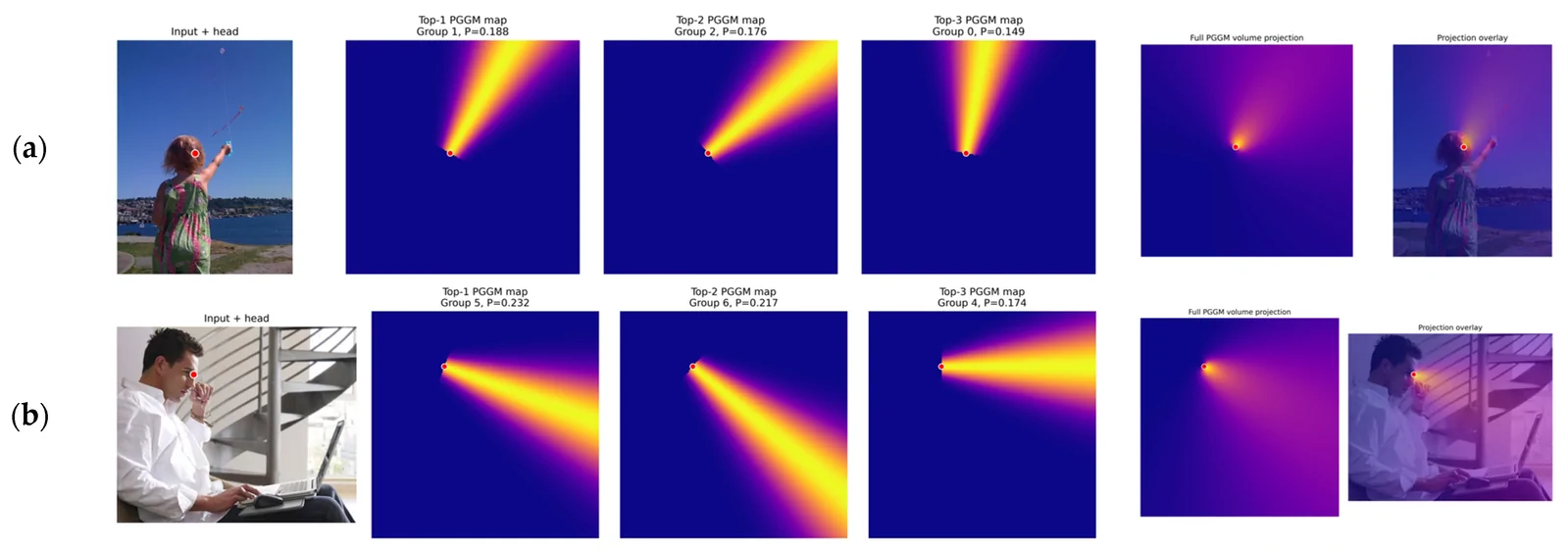
PGGM probability volume visualization. The same two samples as in Figure 3 are used. For each sample, the figure shows the input image with the head position, the top-ranked probability-weighted PGGM maps, and the projection of the full PGGM volume. Sample (**a**) illustrates that PGGM can preserve multiple plausible spatial hypotheses under uncertain gaze direction estimation, while sample (**b**) shows a clearer cone-like spatial prior aligned with the visible head orientation.

To further illustrate how the learned direction distribution is converted into spatial geometric priors, we visualize the PGGM probability volume generated from the same two samples in Figure 4. For each sample, we show the input image with the head position, the top-ranked probability-weighted PGGM maps, and the projection of the full PGGM volume. The top-ranked maps reflect the most likely direction hypotheses, while the full-volume projection aggregates all 18 direction groups and represents the overall spatial prior used by the heatmap network.

For the back-view sample, although the person’s face is not visible, PGGM still produces plausible spatial gaze priors according to the learned direction distribution and the head position. Since the direction prediction is more uncertain in this case, the generated PGGM volume covers a broader set of plausible regions along neighboring directions. This is desirable because a single deterministic direction field may be unreliable when the head is observed from the back. By contrast, the full PGGM volume preserves multiple possible gaze hypotheses and provides the heatmap network with a more flexible spatial prior.

For the side-view sample, the top PGGM maps are more directionally consistent with the visible head orientation. The full PGGM projection forms a clearer cone-like response along the plausible gaze direction, indicating that PGGM successfully transforms the circular direction distribution into an interpretable spatial probability field. Compared with the direct use of a deterministic gaze ray or a fixed-width direction field, the proposed cone-like PGGM can model the gradual expansion of spatial uncertainty along the gaze direction. This helps the network focus on candidate target regions while still preserving reasonable tolerance for direction estimation errors.

Overall, the PGGM visualization confirms the role of PGGM as an intermediate bridge between CDDL and heatmap prediction. CDDL provides uncertainty-aware direction probabilities, while PGGM converts these probabilities into spatial probabilistic gaze maps. The resulting full PGGM volume provides an interpretable direction-aware prior for the final heatmap network.

#### 4.6.3. Heatmap Prediction Visualization and Representative Failure Analysis

**Figure 5:**
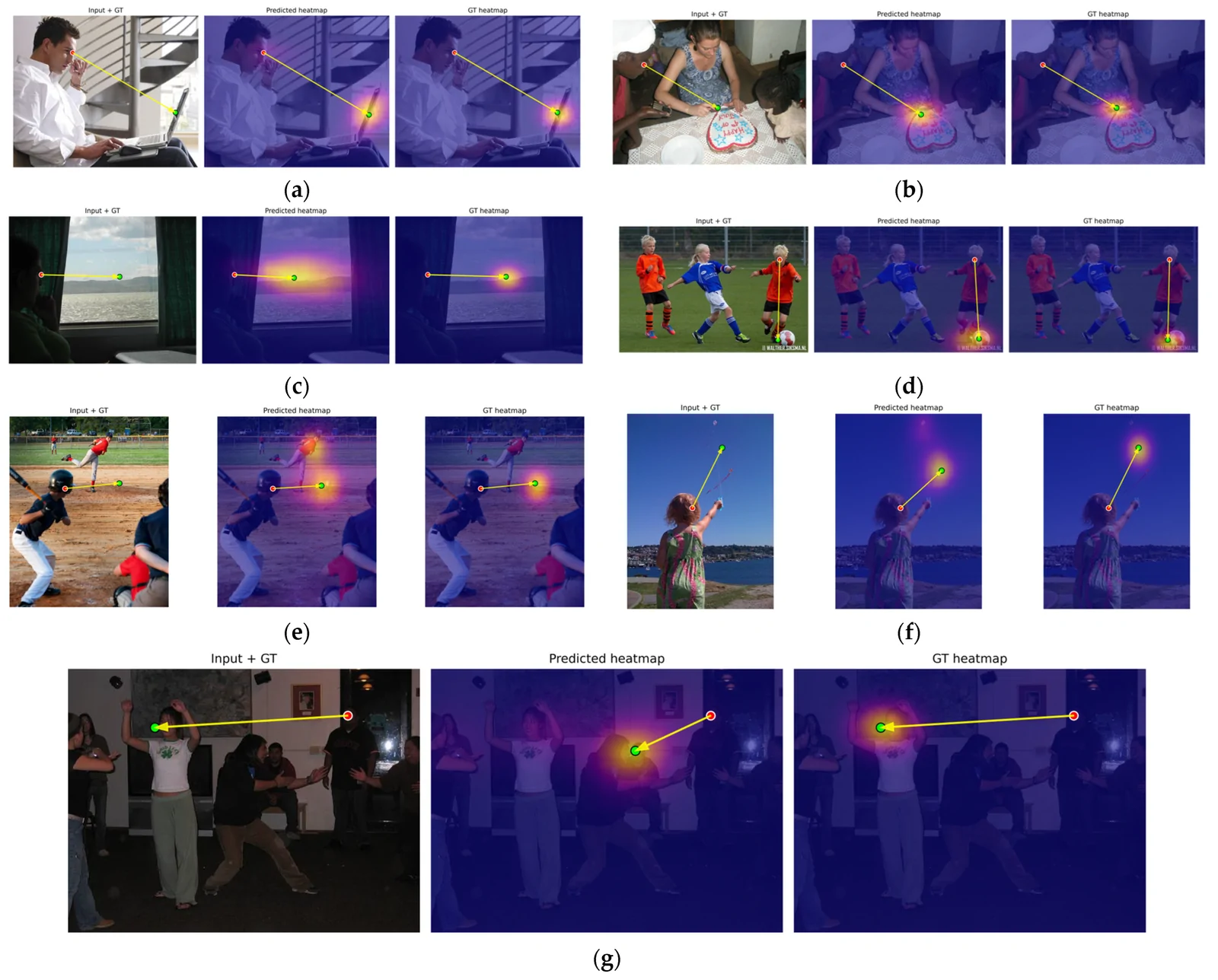
Heatmap prediction visualization on representative samples. Seven qualitative examples are shown. The red dots represent the observer, the green dots represent the observed target, and the yellow arrows represent the gaze direction. Samples (**a**,**b**,**d**) demonstrate accurate gaze target localization under clear visual cues. Sample (**e**) shows that the model can respond to a small moving target in a multi-person sports scene. Samples (**c**,**f**) illustrate that the predicted heatmaps can cover plausible gaze regions under ambiguous annotations and distant small targets. Sample (**g**) shows a challenging low-light multi-person scene where the prediction deviates from the ground truth target, revealing a limitation of the current framework.

To evaluate the final gaze target localization behavior of the proposed framework, we visualize the predicted heatmaps on seven representative samples, as shown in Figure 5. These samples cover a variety of typical gaze-following scenarios, including clear side-view faces, side-back views, low-illumination heads, multi-person sports scenes, back-view subjects, distant small gaze targets, and ambiguous or challenging annotations. Therefore, the visualization not only demonstrates successful localization cases but also reveals how the model behaves under uncertainty and difficult visual conditions.

In Figure 5a,b, the gaze targets are visually clear, and the head orientation provides reliable cues. The predicted heatmaps are highly consistent with the ground truth annotations, showing that the proposed CDDL–PGGM framework can accurately localize gaze targets when the visual evidence is sufficient. Figure 5d shows a multi-person football scene, where the target child is looking at the ball near the feet. Although multiple children appear in the image and face the camera, the predicted heatmap still concentrates around the football, indicating that the proposed method can combine head-centered direction information with scene context to distinguish the actual gaze target from other salient persons.

Figure 5e presents a baseball scene with multiple people and a back-view gaze subject. The ball is flying between the pitcher and the batter. The predicted heatmap produces responses around both the pitcher and the ball, with a stronger activation on the ball, which is consistent with the ground truth gaze target. This example shows that the proposed method can preserve plausible gaze regions along the estimated direction and assign higher confidence to the actual target when the target is small but visually meaningful.

More challenging cases are shown in Figure 5c,f,g. In Figure 5c, the head region is dark, and the gaze target is not a single clearly bounded object. The girl is looking outside the train window toward distant mountains, while the ground truth annotation is placed on one mountain peak. The predicted heatmap covers a wider visible mountain region rather than a single peak. This result is reasonable because the attended region is spatially extended and semantically ambiguous. It also shows that the model can capture the general gaze region even when the exact annotated point is uncertain.

Figure 5f shows a back-view child looking at distant kites. The gaze subject is close to the camera, while the gaze target is small and far away. Moreover, another nearby kite appears close to the annotated gaze target. The predicted heatmap activates around both kites, with a stronger response on the more visually salient kite. Although the peak does not exactly coincide with the annotation, the response still covers plausible gaze target candidates. This case illustrates both the advantage and the limitation of the proposed probabilistic heatmap prediction: the model can preserve multiple reasonable hypotheses under long-range and small-target conditions, but the final peak may still be affected by visual saliency when nearby candidate targets are similar.

Figure 5g represents a difficult low-light indoor scene with multiple interacting people. The target person is relatively far from the camera, and the head region is very dark. The ground truth gaze point lies on the head of a girl closer to the camera, while the predicted heatmap shifts toward another person located between the gaze subject and the annotated target. This failure case suggests that, under severe illumination degradation and complex human interactions, direction-based probabilistic geometry alone may be insufficient to fully resolve the attended person. Explicit object-level or human relation reasoning may further improve localization in such cases.

The examples in Figure 5 are intended to illustrate, rather than replace, the dataset-level error analysis. Cases (c) and (f) exemplify spatially extended annotations and competition between plausible distant targets, whereas case (g) illustrates target selection failure under weak illumination and complex human interactions. Supplementary Tables S5–S8 show that higher heatmap entropy and broader probability mass support are associated with greater annotation dispersion and localization error, and that the highest-error subsets are characterized by broader heatmaps, larger negative log likelihood, and a stronger contribution of radial than lateral error. Figure 5 should therefore be interpreted jointly with the systematic evidence: the probabilistic representation can preserve plausible alternatives, but uncertainty preservation alone does not guarantee correct commitment to one semantic target.

Overall, the heatmap visualization confirms that the proposed framework can generate accurate and interpretable gaze target heatmaps in common cases, while also maintaining reasonable probabilistic responses in ambiguous scenes. The successful cases demonstrate the effectiveness of combining CDDL, Cone-like PGGM, and image-level early fusion. The challenging cases further reveal that the model tends to preserve plausible gaze regions under annotation ambiguity, distant small targets, and weak head cues. At the same time, the observed failure case indicates that future work may benefit from incorporating explicit human–object or human–human relationship modeling.

## 5. Discussion

The experimental results indicate that the proposed framework provides an effective direction-to-region formulation for image-based general gaze following. Rather than treating gaze direction as a single deterministic output, CDDL preserves a distribution of directional hypotheses, and PGGM transforms this distribution into spatial priors for heatmap prediction. The ablation results show that this formulation improves direction estimation relative to direct regression and supports competitive gaze target localization on GazeFollow. In particular, the gains in AUC, minimum distance, and minimum angular error suggest that preserving neighboring directional hypotheses can improve the coverage of plausible gaze targets when the visual evidence does not support one unambiguous direction. This result is consistent with the central motivation of direction-guided gaze following: head-related evidence should constrain scene-based target search, but it should not be assumed to determine a single exact target location in every image [10].

The role of CDDL should be interpreted carefully. Its circular probability distribution represents uncertainty in computational third-person inference, rather than a direct measurement of a person’s oculomotor variability or internal attentional state. In GazeFollow, the target is inferred from image annotations provided by third-person observers, and the annotations may vary when the attended object is small, distant, spatially extended, or ambiguous [9,23]. Under these conditions, a soft directional representation provides a practical way to avoid overconfident supervision. The concentration parameter ablation further supports this interpretation: overly diffuse supervision weakens directional discrimination, whereas overly concentrated supervision approaches hard classification and removes useful neighboring directional information. Therefore, the value of CDDL lies in representing uncertainty that arises from incomplete visual cues and annotation ambiguity, rather than in reproducing a human eye movement distribution.

The PGGM analysis further shows that the way in which directional uncertainty is projected into image space is important. Compared with Strip and Radial formulations, the cone-like PGGM provides the best overall balance across the evaluated metrics. This behavior is geometrically reasonable because an equivalent angular deviation corresponds to a larger positional displacement when the target lies farther from the head. The cone-like field therefore allows spatial support to expand along the predicted direction while maintaining directional selectivity. In addition, the comparison between the Top-3 variant and the full PGGM volume suggests that low-probability direction groups should not be discarded prematurely. Although these groups are individually weak, they can still provide useful contextual support when several objects lie near the same coarse viewing direction.

The fusion experiments also suggest that probabilistic geometry is more useful when it is available during early visual feature extraction. Feature-level fusion introduces the PGGM prior only after the backbone has already formed high-level appearance representations. In contrast, image-level early fusion exposes the backbone to the complete probability volume from the input stage, allowing scene features to be learned conditionally on the directional hypotheses. This result does not imply that early fusion is universally optimal for all gaze-following architectures. Rather, it indicates that, for the present ResNet-50-FPN implementation, early access to the full geometry volume is beneficial for integrating head-related directional evidence with visual scene content.

The qualitative results clarify both the strength and the boundary of the proposed formulation. In clear scenes, the framework produces localized heatmaps that align well with the annotated target. In ambiguous cases, it often retains several plausible response regions instead of collapsing immediately to one isolated location. This behavior is especially relevant for distant small targets, back-view heads, and scenes containing nearby candidate objects. At the same time, the low-light failure case demonstrates that probabilistic direction geometry alone cannot fully resolve complex human interactions or distinguish among semantically related persons. Explicit object-level and human–human relation reasoning, as explored in multi-cue and multi-person gaze-following methods, may provide complementary information in such situations [11,13,19].

The protocol-aware comparison should be interpreted within its stated scope. Under the closest verified conventional evaluator, the proposed model remains competitive, whereas recent Transformer and foundation–encoder methods report stronger AUC under different modern evaluation settings. The contribution of CDDL–PGGM is therefore established primarily by the matched direction, geometry, and fusion analyses, which support an explicit direction space uncertainty representation and its channel-wise projection before scene-based target selection.

From a broader social-attention perspective, preserving multiple plausible direction-to-region hypotheses is conceptually compatible with shared-attention accounts in which gaze perception contributes to the coordination of attention and higher-level social cognition [4]. Evidence that facial cues influence overt orienting and saccadic trajectories also underscores the behavioral relevance of gaze-related signals [8]. However, CDDL–PGGM is not a cognitive model of joint attention, nor does its learned distribution measure human perceptual or oculomotor variability. It is a computational representation of uncertainty in third-person gaze target inference under incomplete visual evidence.

Several limitations should be acknowledged. The framework operates on single images and does not exploit temporal continuity. It also lacks explicit object categories, object proposals, and human–human relation representations, which may be important when several semantically plausible targets lie along similar directions. Zero-shot VAT evaluation broadens the empirical context, while the coverage-limited ChildPlay analysis shows that the incremental benefit of the probabilistic volume is domain-dependent. The learned distribution has not been validated against synchronized eye tracking or inter-observer attentional data and should therefore not be interpreted as human uncertainty. Finally, the complete Direction Probability Volume introduces non-trivial latency and memory costs despite a small parameter increase. Future work should investigate stronger visual encoders, object-level semantic relations, distance-aware target commitment, temporal evidence, psychophysical validation, and lightweight or adaptive direction channel selection.

Overall, the findings support the use of a circular direction distribution and explicit geometric projection as a transparent bridge between head-related evidence and scene-based target localization. The supported benefits are measurable but not uniform across metrics or domains, and the representation does not replace semantic, relational, or temporal reasoning. Its value lies in preserving interpretable directional alternatives that can be combined with those complementary sources of evidence.

## 6. Conclusions

This study introduced an uncertainty-aware framework for image-based general gaze following based on Circular Direction Distribution Learning and Probabilistic Gaze Geometry Modeling. CDDL represents uncertainty in circular direction space, and PGGM projects the retained hypotheses into a channel-wise Direction Probability Volume before image-level fusion and heatmap prediction. Controlled experiments, zero-shot transfer, and uncertainty analyses support the usefulness of this explicit direction-to-region representation while also revealing domain-dependent and computational trade-offs. The framework is therefore best viewed as an interpretable complement to stronger semantic, relational, and temporal reasoning for gaze target disambiguation.

## Figures and Tables

**Table 1:** The detailed hardware and software environment.

Component	Specification
CPU	Intel Core i7-12700KF @ 3.60 GHz
GPU	NVIDIA GeForce RTX 5090 D v2, 24 GB VRAM
Memory	64 GB DDR4 3200 MHz (4 × 16 GB Corsair)
Operating System	Windows 11
Python	Python 3.11.14
Deep Learning Framework	PyTorch 2.12.0
CUDA Toolkit	CUDA 13.0.1
cuDNN	cuDNN 9.2.0

**Table 2:** Effect of the von Mises concentration parameter and learning rate schedule on CDDL direction prediction.

κ	Scheduler	Top-1 (%) ↑	Top-3 (%) ↑	Top-5 (%) ↑	Dir-Ang (°) ↓	Dir-MAng (°) ↓
2	Fixed LR	11.00	32.50	49.04	20.20	11.15
3	Fixed LR	12.09	34.21	50.56	19.70	10.80
4	Fixed LR	11.86	34.78	51.23	19.69	10.92
4	StepLR	12.09	35.38	52.40	19.03	10.44
5	Fixed LR	11.84	34.09	51.07	20.18	11.23
6	Fixed LR *	11.42	34.61	51.38	19.72	10.80
**6**	**StepLR** *	**12.92**	**37.03**	**53.70**	**18.72**	**9.90**
8	Fixed LR	12.00	33.50	50.73	19.93	11.03

* The upward arrow indicates that the higher the value of this indicator, the better, while the downward arrow indicates the opposite. All the tables in this paper adopt this representation. Fixed LR denotes fixed learning rate training. StepLR denotes learning rate decay using a step scheduler. Each row reports official GazeFollow test-set performance for the model selected on validation Dir-Ang. The final κ = 6 StepLR configuration was chosen from validation evidence before test evaluation. Bold indicates the adopted setting rather than a claim of theoretical optimality.

**Table 3:** Controlled comparison between deterministic direction regression and CDDL.

Method	Schedule	Top-1 *(%) ↑	Top-3 *(%) ↑	Top-5 *(%) ↑	Dir-Ang (°) ↓	Dir-MAng (°) ↓
One Human [9] *	-	-	-	-	11.00	-
Direction Regression	Fixed LR	-	-	-	22.74	13.32
CDDL (κ=6)	Fixed LR	11.42	34.61	51.38	19.72	10.80
**CDDL (κ=6)**	**StepLR**	**12.92**	**37.03**	**53.70**	**18.72**	**9.90**

* Top-k Accuracy is defined only for the distribution-based CDDL output. The fixed learning rate comparison controls the learning rate schedule, while the StepLR row corresponds to the final selected direction model. One Human is reported as a human-level reference. Bold indicates the adopted setting rather than a claim of theoretical optimality.

**Table 4:** Controlled comparisons of Probabilistic Direction Field formulation and direction group count on the held-out validation subset.

(a). Probabilistic Direction Field formulation.			
Formulation	AUC ↑	Dist ↓	BCE ↓
GSF	0.926686	0.154466	0.059974
RDGF	0.924158	0.157468	0.060581
**CGF**	**0.925567**	**0.154216**	**0.060216**
**(b)** **. Number of direction groups.**			
**Direction Groups**	**AUC ↑**	**Dist ↓**	**BCE ↓**
9	0.924986	0.154391	0.060268
**18**	**0.925567**	**0.154216**	**0.060216**
36	0.925518	0.156557	0.060988

Note: All values were computed on the held-out validation subset derived from the official training split. AUC is per-image AUC at the prediction heatmap resolution, and BCE is mean binary cross-entropy loss. Bold identifies the retained configuration, not a theoretically unique optimum.

**Table 5:** Sensitivity of Cone-like Gaussian Field to the cone base width on the held-out validation subset.

Cone Base Width $\sigma_{0}$ (Pixels)	AUC ↑	Dist ↓	BCE ↓
2	0.925456	0.154032	0.060181
4	0.924975	0.153709	0.060282
6	0.925567	0.154216	0.060216
8	0.925471	0.154329	0.060312
**10**	**0.925955**	**0.154185**	**0.060163**

Note: Metric definitions follow Table 4. All variants use image-level Full-18 fusion with 18 direction groups. Bold identifies the validation-selected operating point; the lowest Dist was obtained with a base width of 4 pixels.

**Table 6:** Controlled comparison of PGGM fusion strategies on the held-out validation subset.

Fusion Strategy	Direction Prior	AUC ↑	Dist ↓	BCE ↓
Feature-level fusion *	Full-18 PGGM volume	0.924716	0.162475	0.061570
Image-level early fusion *	Top-3 PGGM maps	0.925747	0.154251	0.060076
**Image-level early fusion**	**Full** **-18** **PGGM volume**	**0.925567**	**0.154216**	**0.060216**

* Feature-level fusion denotes concatenating the PGGM volume with the FPN feature map, while image-level early fusion denotes concatenating PGGM maps with the RGB image before the ResNet-FPN heatmap network. Metric definitions follow Table 4. All variants use CGF with a base width of 6 pixels for the controlled fusion comparison. Bold identifies the representation retained in the proposed framework.

**Table 7:** Protocol-aware comparison with representative gaze-following methods on GazeFollow.

Historical benchmark baselines and human reference.							
Method			AUC ↑	Dist ↓	MDist ↓	Ang ↓	MAng * ↓
Random [9]			0.504	0.484	0.391	69.0°	-
Center [9]			0.633	0.313	0.230	49.0°	-
Fixed bias [9]			0.674	0.306	0.219	48.0°	-
Judd et al. [29]			0.711	0.337	0.250	54.0°	-
SalGAN [30]			0.848	0.238	0.192	36.7°	22.4°
Recasens et al. [9]			0.878	0.190	0.113	24.0°	-
Recasens et al. (ResNet) [10] *			0.881	0.175	0.101	22.5°	11.6°
One Human [9]			0.924	0.096	0.040	11.0°	-
**(a)** **. Strict conventional pooled/global comparison.**							
**Method**			**AUC** **↑**	**Dist** **↓**	**MDist** **↓**	**Ang** **↓**	MAng * ↓
Lian et al. [10]			0.9060	0.1450	0.0810	17.60	8.80
**Ours**			**0.9066**	**0.1423**	**0.0801**	**16.90**	**8.23**
**(b)** **. Modern per-image or per-person-instance AUC.**							
**Method**	**Reported** **AUC** **↑**	**Verified Evaluator Family**			**Representation/Model Class**		
Chong et al. [57]	0.921	Original resolution per-image mean			Scene–head heatmap model		
PDP [23]	0.934	Original resolution per-image mean			Patch-level target distribution		
Sharingan [13]	0.944	Per-image mean; output resolution			Multi-person Transformer		
ViTGaze [14]	0.949	Original resolution per-image mean			Transformer interaction features		
Gaze-LLE, ViT-L [15]	0.958	Original resolution per-person-instance mean			Large frozen visual encoder		
Ours	0.9214	Original-resolution per-image mean			ResNet-50 direction-to-region model		
**(c)** **. Methods using additional cues, modalities, or training resources.**							
**Method**		**Reported** **AUC** **↑**	**Main Comparability Difference**				
DAM [58]		0.922	Monocular depth and explicit three-dimensional gaze cues				
ESCNet [59]		0.928	Three-dimensional geometry and scene-understanding modules				
Gupta et al., image-only [60]		0.933	Additional gaze direction pretraining on Gaze360				
Gupta et al., multimodal [60]		0.943	RGB, depth, pose, and additional pretraining				
Head–Local–Global Coordination [61]		0.939	Local/global coordination, depth, and multiple gaze-pretraining datasets				

* Recasens et al. (ResNet) denotes the ResNet-50-based reimplementation reported in [10]. MAng is only available for methods that report this metric. The exact spatial resolution and cross-sample aggregation of the historical Recasens AUC implementation could not be reconstructed from the paper alone. Table 7a restricts direct AUC comparison to the verified conventional pooled/global protocol. Values in Table 7b,c are reported for research positioning and are not treated as interchangeable with Table 7a. Bold identifies the proposed model in Table 7a.

**Table 8:** Zero-shot cross-dataset evaluation on the in-frame portion of the official VAT test split.

Model	AUC ↑	VAT Distance ↓	Short-Side Dist ↓	Ang (°) ↓
RGB-only	0.816993	0.271442	0.405192	36.30
Deterministic CGF	0.873652	0.166578	0.235087	20.33
Proposed Full-18 CGF	0.882292	0.157374	0.226388	19.48

Note: AUC and VAT Distance follow the VAT benchmark-style in-frame evaluation. Short-side Dist normalizes peak localization error by the shorter image side, and Ang is computed from the person location to the predicted and annotated gaze targets. All rows use the same GazeFollow-trained models and the same VAT evaluation set.

**Table 9:** Computational efficiency of representative model configurations.

Model	Input Channels	Inference-Active Parameters (M)	Batch-1 Latency (ms/Sample)	Batch-64 Throughput (Samples/s)	Peak Allocated Memory (GB)
RGB-only	3	29.27	3.85 (0.14)	2295.36 (1.41)	1.42
Deterministic CGF	4	54.05	9.52 (0.10)	1313.55 (0.72)	1.52
Proposed Full-18 CGF	21	54.10	12.54 (0.08)	1143.48 (16.96)	2.19

Note: Inference-active parameters include only modules executed in the corresponding forward path; the RGB-only model bypasses the direction-estimation branch. Latency and throughput are mean (standard deviation) over five repetitions. Peak memory denotes CUDA maximum allocated memory during forward inference after the model and fixed input tensors were resident on the GPU.

## Data Availability

Data are available from the corresponding author upon reasonable request.

## Supplementary Materials

The following supporting information can be downloaded at: https://www.mdpi.com/article/10.3390/jemr19040088/s1, Methods S1: Separation of validation-based model selection and final test evaluation; Methods S2: External zero-shot evaluation protocol; Methods S3: Computational-efficiency benchmarking protocol; Methods S4: Internal-uncertainty and error-characterization protocol; Table S1: GazeFollow test set results for the principal controlled model variants; Table S2: Paired effect estimates for Proposed Full-18 CGF relative to Deterministic CGF on VAT; Table S3: Zero-shot results on the coverage-limited available ChildPlay test subset; Table S4: Full computational-efficiency results; Table S5: Rank associations among annotation dispersion, predictive uncertainty, and localization error; Table S6: Localization and annotation statistics across predictive-uncertainty quintiles; Table S7: Deterministic and probabilistic performance across annotation-dispersion strata; Table S8: Aggregate profiles of the highest-error subsets; Supplementary Note S1: Interpretation of the controlled PGGM analyses; Supplementary Note S2: Cross-dataset interpretation; Supplementary Note S3: Interpretation of internal uncertainty reliability and failure profiles.

## Abbreviations

The following abbreviations are used in this manuscript:

GT	Ground truth
CDDL	Circular Direction Distribution Learning
PGGM	Probabilistic Gaze Geometry Modeling
FPN	Feature Pyramid Network
BCE	Binary Cross-Entropy
AUC	Area Under the Curve
