# Supplementary material for: Uncertainty-Aware General Gaze Following via Circular Direction Distribution Learning and Probabilistic Gaze Geometry Modeling
Source: J Eye Mov Res. 2026 Aug 13;19(4):88. doi: 10.3390/jemr19040088 (PMC13514171; doi:10.3390/jemr19040088)
Supplement: Supplementary file 1 [file jemr-19-00088-s001.zip › jemr-4430111-supplementary.pdf]

Supplementary Materials

Uncertainty-Aware General Gaze Following via Circular Direction Distribution Learning and Probabilistic Gaze Geometry Modeling

Yanzhao Li, Jin Li, Xiaona Zhang, and Hong Liang*

** Correspondence: lh_hrbeu@163.com*

Methods S1. Separation of Validation-Based Model Selection and Final Test Evaluation

The GazeFollow experiments used a held-out validation subset derived from the official training split for model and hyperparameter selection. Annotations were grouped by image identity before partitioning so that person instances from the same image could not occur in both the training and validation subsets.

The Probabilistic Direction Field formulation, direction group count, cone base width, fusion strategy, and training epoch were selected using only this held-out validation subset. The official GazeFollow test set was reserved for final evaluation. The validation tables in the main manuscript therefore report the quantities used for model selection, whereas the test set results below provide a separate assessment of the selected and representative controlled variants.

Each validation instance contains a single gaze annotation. Consequently, MDist is identical to Dist, and MAng is identical to Ang on the validation subset. The model selection tables report per-image AUC at the prediction-heatmap resolution, Dist, and mean binary cross-entropy loss. The final test evaluation uses all valid annotations and the complete metric protocol defined in Section 4.1.2 of the main manuscript.

Table S1. GazeFollow test set results for the principal-controlled model variants.

Model	Pooled/global AUC ↑	Original-resolution per-image AUC ↑	Dist ↓	MDist ↓	Ang (°) ↓	MAng (°) ↓
RGB-only	0.8501	0.8853	0.2096	0.1415	31.42	20.39
Deterministic CGF	0.9005	0.9178	0.1468	0.0845	16.98	8.10
Image-level Top-3 CGF	0.9048	0.9210	0.1438	0.0821	16.92	8.14
Image-level Full-18 CGF, base width 6	0.9060	0.9212	0.1424	0.0806	16.89	8.24
**Image-level Full-18 CGF, base width 10**	**0.9066**	**0.9214**	**0.1423**	**0.0801**	**16.90**	**8.23**

Note: The pooled/global AUC follows the conventional low-resolution protocol used for the historical comparison, whereas the original-resolution AUC is computed per image and then averaged. All localization and angular metrics use every valid annotation. Bold identifies the final proposed configuration.

Supplementary Note S1. Interpretation of the Controlled PGGM Analyses

Field formulation. Across the final test metrics, no single formulation achieved a stable all-metric advantage among GSF, CGF, and RDGF. The combined validation and test evidence therefore supports interpreting the formulations as a trade-off between discrimination and localization rather than as a universal ranking.

Direction group count. The 18-group and 9-group representations were not statistically distinguishable on the principal paired test metrics. Relative to 36 groups, the 18-group representation retained a similar AUC with a lower localization distance and fewer direction-field channels.

Cone base width. The base-width-6 and base-width-10 variants remain close on the official test set: increasing the base width from 6 to 10 raises pooled/global AUC from 0.9060 to 0.9066 and reduces MDist from 0.0806 to 0.0801, whereas the base-width-6 variant has a marginally lower Ang (16.89° versus 16.90°). No pair among the evaluated base widths showed a difference that remained supported after Holm correction across the principal test metrics. A base width of 10 pixels is therefore retained because it was selected on the held-out validation subset, not because the test results establish a universal optimum.

Fusion strategy. Image-level Full-18 fusion reduced Dist, MDist, Ang, and MAng relative to feature-level Full-18 fusion, whereas the per-image AUC difference was not statistically supported. Image-level Top-3 and Full-18 were not statistically distinguishable on the principal test metrics. Full-18 is retained for its complete direction group representation.

Methods S2. External Zero-Shot Evaluation Protocol

The RGB-only, deterministic CGF, and proposed Full-18 CGF models used in the external evaluation were trained exclusively on GazeFollow. Neither VAT nor ChildPlay was used for training, fine-tuning, model selection, or configuration adjustment.

For VAT, the evaluation used all 20,655 in-frame person frames in the official test split. The present model does not include an out-of-frame classification branch; therefore, out-of-frame average precision was not evaluated. AUC and VAT Distance follow the benchmark-style in-frame implementation. Short-side Dist normalizes the Euclidean localization error by the shorter image side, and Ang measures the angular difference between the predicted and annotated head-to-target directions.

For ChildPlay, only the coverage-limited available test inside-visible subset could be evaluated because the locally available media covered 25 of the 45 official test clips. The subset contains 14,332 person frames. These results are reported only as a coverage-limited generalization analysis and are not compared directly with complete benchmark results from other methods.

Table S2. Paired effect estimates for Proposed Full-18 CGF relative to Deterministic CGF on VAT.

Metric	Mean change	Bootstrap 95% CI	Paired evidence	Interpretation
AUC	+0.008640	[+0.007795, +0.009444]	Holm-adjusted paired test supported	Improved distribution-level discrimination
VAT Distance	−0.009204	[−0.010964, −0.007578]	Wilcoxon p = 0.375	Lower mean; rank-based evidence mixed
Short-side Dist	−0.008699	[−0.011033, −0.006228]	Holm-adjusted paired test supported	Improved peak localization
Ang	−0.852°	[−1.047°, −0.654°]	Holm-adjusted paired test supported	Improved directional localization

Note: Changes are Proposed Full-18 CGF minus Deterministic CGF; negative distance and angular changes indicate improvement. The VAT Distance mean is lower, but its paired rank-based evidence is mixed.

Table S3. Zero-shot results on the coverage-limited available ChildPlay test subset.

Group / model	AUC ↑	Dist ↓	Short-side Dist ↓	Ang (°) ↓
Full / RGB-only	0.925173	0.142534	0.189235	16.84
Full / Deterministic CGF	0.934779	0.122500	0.159460	12.51
Full / Proposed Full-18 CGF	0.936229	0.133207	0.169686	13.24
Child / Deterministic CGF	0.956173	0.089996	0.119275	10.71
Child / Proposed Full-18 CGF	0.957150	0.101622	0.130236	11.57
Adult / Deterministic CGF	0.910045	0.160080	0.205921	14.60
Adult / Proposed Full-18 CGF	0.912041	0.169725	0.215297	15.17

Note: The available subset contains 14,332 inside-visible person frames from 25 of the 45 official test clips. It is not a complete ChildPlay benchmark. Proposed Full-18 CGF produces a small AUC increase over deterministic CGF but does not improve peak-based localization errors.

Supplementary Note S2. Cross-Dataset Interpretation

Across VAT and the available ChildPlay subset, the most stable cross-dataset finding is the benefit of explicit head-centered gaze geometry relative to RGB-only prediction. The incremental benefit of retaining multiple probabilistic direction channels is domain-dependent: it improves AUC, short-side distance, and angular error on VAT, whereas on the available ChildPlay subset it provides only a small AUC gain and worsens peak localization. The cross-dataset evidence therefore supports a qualified generalization claim rather than uniform probabilistic superiority.

Methods S3. Computational-Efficiency Benchmarking Protocol

Computational efficiency was measured using the RGB-only model, the deterministic CGF model, and the proposed Full-18 CGF model. All measurements were performed on an NVIDIA GeForce RTX 5090 D v2 GPU using PyTorch 2.12.0, CUDA 13.0, the PyTorch runtime cuDNN 9.2.0, and FP32 inference. The input and output resolutions were 224 × 224 and 56 × 56, respectively.

Fixed synthetic tensors were used to isolate model forward cost from image decoding, storage access, DataLoader behavior, and host-to-device transfer. The timing scope included only the model forward pass and excluded data loading, host-to-device transfer, metric computation, and CPU post-processing. cuDNN benchmarking was enabled.

For batch size 1, each model was warmed up for 50 iterations and then timed for 200 iterations per repetition. For batch size 64, each model was warmed up for 20 iterations and timed for 50 iterations per repetition. Five repetitions were performed for each configuration. CUDA synchronization was applied immediately before and after each timed block.

Peak allocated and peak reserved GPU memory were recorded after the model and fixed input tensors were resident on the GPU. Inference-active parameters include only modules executed in the corresponding forward path; the RGB-only model bypasses the direction estimation branch.

Table S4. Full computational-efficiency results.

Model	Batch size	Latency (ms/sample)	Throughput (samples/s)	Peak allocated memory (GB)	Peak reserved memory (GB)	Inference-active parameters (M)
RGB-only	1	3.854 (0.136)	259.73 (9.12)	0.265	0.287	29.270
RGB-only	64	0.436 (0.000)	2295.36 (1.41)	1.424	1.959	29.270
Deterministic CGF	1	9.521 (0.099)	105.03 (1.09)	0.300	0.321	54.048
Deterministic CGF	64	0.761 (0.000)	1313.55 (0.72)	1.522	1.992	54.048
Proposed Full-18 CGF	1	12.539 (0.078)	79.75 (0.50)	0.310	0.350	54.101
Proposed Full-18 CGF	64	0.875 (0.013)	1143.48 (16.96)	2.191	2.833	54.101

Note: Latency and throughput values are reported as mean (standard deviation) over five repetitions. Memory values use decimal gigabytes. The benchmark measures model-forward inference only.

Methods S4. Internal-Uncertainty and Error-Characterization Protocol

The analysis uses the 4,782 instances in the official GazeFollow test set and the final Image-level Full-18 CGF model with a cone base width of 10 pixels. Direction entropy is computed from the normalized circular direction distribution. Heatmap entropy is computed from the normalized predicted heatmap, and the 90% mass area is the fraction of heatmap pixels required to accumulate 90% of the normalized probability mass.

Annotation dispersion is the mean Euclidean distance between the valid human annotations and their mean position in normalized image coordinates. Associations are summarized using Spearman rank correlation. Reliability is further examined by dividing direction entropy and heatmap entropy into five equal-frequency groups. Annotation dispersion strata contain equal-size low, medium, and high groups.

High-error profiles are reported for the 100 largest final model Dist values and for the 100 samples with the largest increase in Dist relative to deterministic CGF. These sets are used for aggregate error characterization and not for model selection. Because GazeFollow does not provide synchronized subjective confidence or psychophysical uncertainty labels, the analysis is described as internal uncertainty reliability rather than human uncertainty calibration.

Table S5. Rank associations among annotation dispersion, predictive uncertainty, and localization error.

Variable 1	Variable 2	N	Spearman correlation	p-value
Annotation dispersion	Direction entropy	4782	0.146	2.57e-24
Annotation dispersion	Heatmap entropy	4782	0.426	1.72e-210
Annotation dispersion	90%-mass area	4782	0.413	7.66e-197
Annotation dispersion	Dist	4782	0.499	6.81e-300
Direction entropy	Dist	4782	0.189	1.56e-39
Heatmap entropy	Dist	4782	0.498	1.77e-298
Direction entropy	Heatmap entropy	4782	0.326	4.40e-119

Note: Correlations are descriptive associations across the complete GazeFollow test set. They do not constitute psychophysical calibration.

Table S6. Localization and annotation statistics across predictive uncertainty quintiles.

Uncertainty measure	Quintile	N	Mean uncertainty	Dist	Ang (°)	Annotation dispersion	Multi-annotation NLL
Direction entropy	Q1	957	0.721	0.1010	7.14	0.0607	5.710
Direction entropy	Q2	956	0.740	0.1307	10.55	0.0729	6.037
Direction entropy	Q3	956	0.757	0.1383	13.72	0.0766	6.197
Direction entropy	Q4	956	0.780	0.1592	18.91	0.0834	6.423
Direction entropy	Q5	957	0.861	0.1823	34.17	0.0896	6.685
Heatmap entropy	Q1	957	0.693	0.0537	8.05	0.0418	5.147
Heatmap entropy	Q2	956	0.748	0.1041	13.54	0.0610	5.915
Heatmap entropy	Q3	956	0.785	0.1426	16.39	0.0770	6.297
Heatmap entropy	Q4	956	0.818	0.1716	19.96	0.0955	6.624
Heatmap entropy	Q5	957	0.864	0.2393	26.56	0.1078	7.067

Note: Q1 denotes the lowest uncertainty quintile and Q5 the highest. Each uncertainty measure is stratified independently.

Table S7. Deterministic and probabilistic performance across annotation dispersion strata.

Panel A. Localization metrics.

Model	Dispersion group	N	Per-image AUC	Dist	MDist	Ang (°)	MAng (°)
Deterministic CGF	low	1594	0.9679	0.0913	0.0713	12.52	8.36
Deterministic CGF	medium	1594	0.9313	0.1373	0.0843	15.69	7.62
Deterministic CGF	high	1594	0.8541	0.2118	0.0978	22.73	8.31
**Proposed Full-18 CGF**	**low**	**1594**	**0.9704**	**0.0872**	**0.0675**	**12.34**	**8.31**
**Proposed Full-18 CGF**	**medium**	**1594**	**0.9352**	**0.1316**	**0.0818**	**15.46**	**7.72**
**Proposed Full-18 CGF**	**high**	**1594**	**0.8587**	**0.2080**	**0.0933**	**22.90**	**8.68**

Panel B. Predictive spread for the proposed model.

Dispersion group	N	Annotation dispersion	Heatmap entropy	90%-mass area	Multi-annotation NLL
low	1594	0.0229	0.751	0.142	5.381
medium	1594	0.0590	0.781	0.174	6.118
high	1594	0.1479	0.812	0.223	7.131

Note: The proposed model retains slightly higher AUC and lower Dist/MDist than deterministic CGF in all three dispersion strata, whereas the angular differences are not uniform.

Table S8. Aggregate profiles of the highest-error subsets.

Panel A. Uncertainty and localization profile.

Subset	N	Per-image AUC	Dist	Ang (°)	Heatmap entropy	90%-mass area	Annotation dispersion	NLL
All test instances	4782	0.921	0.142	16.90	0.782	0.180	0.077	6.210
Top 100 by final Dist	100	0.719	0.643	62.02	0.841	0.283	0.083	8.445
Top 100 by final-minus-deterministic Dist	100	0.869	0.458	34.59	0.840	0.264	0.080	7.028

Panel B. Radial–lateral error decomposition.

Subset	Mean radial absolute error	Mean lateral absolute error	Radial > lateral	Mean final-minus-deterministic Dist
All test instances	0.107	0.069	60.2%	-0.005
Top 100 by final Dist	0.485	0.272	69.0%	0.131
Top 100 by final-minus-deterministic Dist	0.358	0.195	78.0%	0.349

Note: The high-error subsets show broader heatmaps, higher negative log-likelihood, and a stronger radial contribution than the complete test set. The final-minus-deterministic subset is defined only for diagnostic comparison and was not used for model selection.

Supplementary Note S3. Interpretation of Internal Uncertainty Reliability and Failure Profiles

Direction entropy, heatmap entropy, and probability mass area capture related but non-identical aspects of uncertainty. Direction entropy is only weakly related to annotation dispersion, suggesting that ambiguity in the inferred head-centered direction is not sufficient to explain inter-annotator variability. Heatmap entropy and 90% mass area show stronger associations with annotation dispersion because they also reflect scene-level target ambiguity and the spatial extent of

plausible targets. Their monotonic relationship with Dist and Ang supports their use as indicators of internal error risk. However, these quantities should not be interpreted as calibrated human confidence. The high-error profiles further show that severe failures are not explained solely by diffuse probability maps: radial misplacement, long-range target commitment, visual saliency, and human–object or human–human relations remain important limitations.
